# Iodine/DMSO-catalyzed oxidative deprotection of *N*-tosylhydrazone for benzoic acid synthesis†

**DOI:** 10.1039/d4ra05849f

**Published:** 2024-09-24

**Authors:** Rakshanda Singhal, Manish K. Mehra, Babita Malik, Meenakshi Pilania

**Affiliations:** a Department of Chemistry, Manipal University Jaipur Jaipur (Rajasthan) VPO-Dehmi-Kalan, Off Jaipur-Ajmer Express Way Jaipur Rajasthan 303007 India meenakshi.pilania@jaipur.manipal.edu; b Department of Chemistry, Birla Institute of Technology and Science Pilani Pilani Campus Rajasthan 333031 India

## Abstract

An oxidative deprotection of tosylhyrdazones has been demonstrated to afford benzoic acids using iodine and DMSO system. This efficient oxidative deprotection protocol offers exceptional functional group toleration under mild reaction conditions without any initiators or bases. Notably, the tosylhydrazone with the heteroaryl ring or with the aryl ring having base-sensitive hydroxyl and ester functional groups smoothly afforded the corresponding benzoic acid analogues under developed conditions. Moreover, this method features short reaction times, high product yields and easy purification by avoiding column-chromatographic purification.

## Abstract 2

An oxidative deprotection approach has been developed using iodine-DMSO system to synthesize carboxylic acid derivatives from *N*-tosylhydraones, yielding good to excellent results.

## Graphical abstract

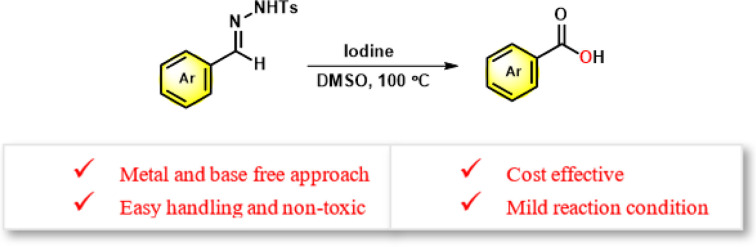

## Introduction

*N*-tosylhydrazones are popular synthons in synthetic organic chemistry for constructing potent cyclic scaffolds.^1,2^ The synthesis of tosylhydrazones can be straightforwardly achieved in solid form *via* condensation of carbonyl compounds with tosylhydrazine in high yields.^3,4^ However, ascribed to their appealing features and potent diazo and 1,3-dipolar precursors, tosylhydrazones have played a crucial role in the synthesis or modification of various bioactive heterocycles.^5–10^ Additionally, due to their crystalline and stable nature, tosylhydrazones and its derivatives have been used as protection and purification agents for carbonyl compounds.^11–14^ The deprotection of hydrazones into corresponding carbonyl or carboxylic acid derivatives under mild conditions is a vital process in organic synthesis. The development of mild and effective methods for the deprotection of procarbonyl compounds has been of long-standing interest of organic chemists. To date, numerous conditions or catalysts such as copper(i) chloride,^15^ clayfen,^16^ potassium bromate,^17^ quinolinium dichromate (QDC),^18^ alumina-supported ammonium chlorochromate,^19^ 6-benzyl-4-aza-1-azoniabicyclo[2.2.2]octane dichromate,^20^ and Amberlyst 15 supported nitrosonium ion^21^ have been reported to accomplish the deprotection of hydrazones into corresponding carbonyl compounds. Although multiple approaches for the regeneration of carbonyl compounds from oximes^11,22,23^ are reported in scientific literature, only a few reports are available from tosylhydrazones, especially with mild reaction conditions.

**Scheme 1:**
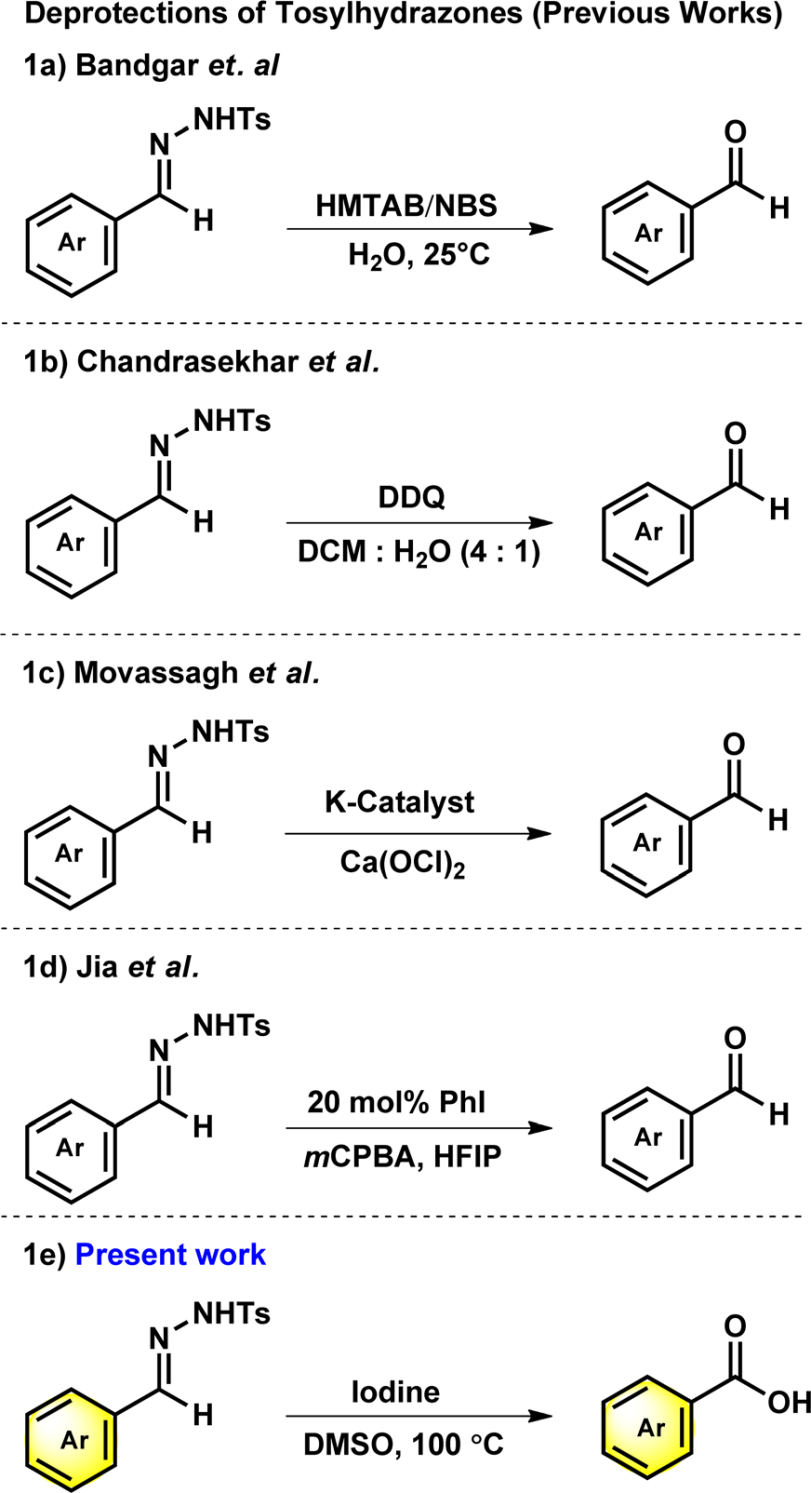
Deprotection of *N*-tosylhydrazones.

**Fig. 1:**
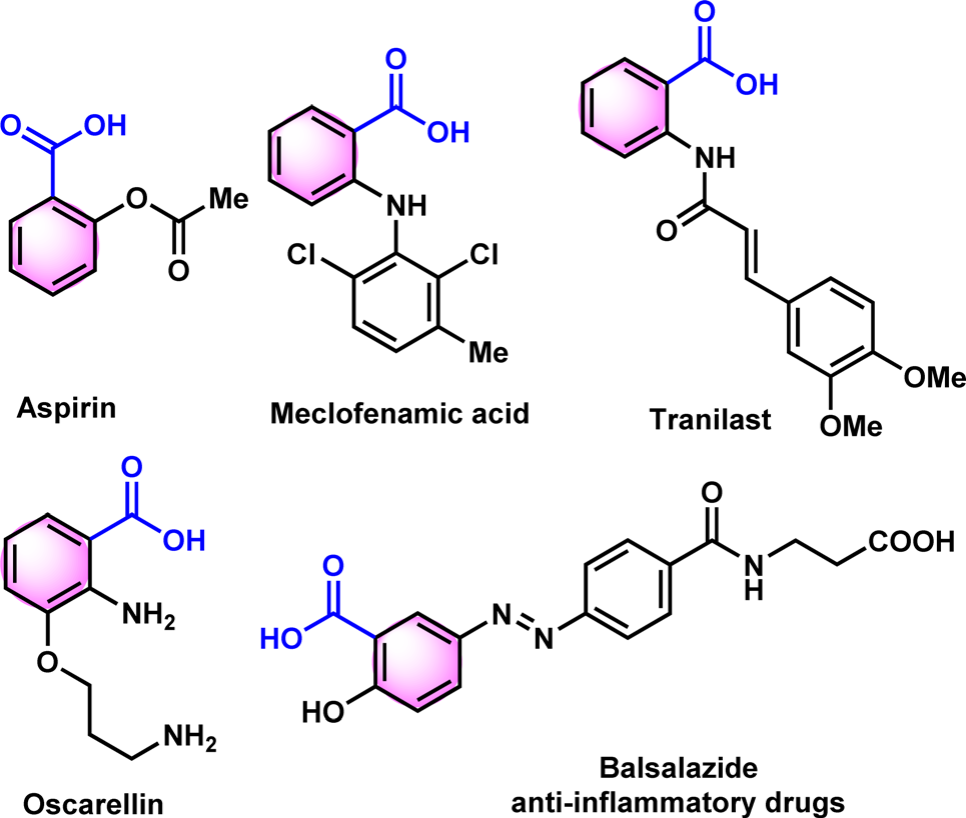
Representative biologically active benzoic acids.

In 2000, Bandgar and his team reported the regeneration of carbonyl compounds using hexamethylenetetramine–bromine (HMTAB) and *N*-bromosuccinimide (NBS) (Scheme 1a).^24^ Similarly, in the same year, Chandrasekhar and his team disclosed the selective cleavage of tosylhydrazone using 2,3-dichloro-5,6-dicyano-1,4-benzoquinone (DDQ) (Scheme 1b).^25^ In 2006, Movassagh and co-workers utilized K-catalyst and calcium hypochlorite for the deprotection of tosylhydrazones (Scheme 1c).^26^ Likewise, Jia and his group regenerated the carbonyl compounds using *meta*-chloroperbenzoic acid (*m*CPBA) (Scheme 1d).^27^ Despite many attempts thus far, existing methods or conditions often suffer from various disadvantages *e.g.* expensive syntheses, limited substrate scope, extended reaction time, and the use of hazardous oxidants and metal ions in certain cases. Noticeably, the usage of a mild oxidation approach for the direct conversion of tosylhydrazones to corresponding carboxylic acids is still desirable. It is therefore necessary to develop new methods based on readily available oxidants and safer chemicals. In this context, iodine and DMSO system have recently received attention as mild and selective oxidizing agent. The I_2_/DMSO duo is very suitable for chemoselective oxidation, dehydrogenation, oxidative aromatization, protection/deprotection of various functional groups, and regioselective and stereoselective transformations.^28–30^ On the other hand, the selective oxidation of carbonyl groups is a vital step in organic synthesis since it enables the production of acids, amides, acetals, and esters.^31–38^ Carboxylic acids are the most extensively used oxidized feedstocks and are needed in large amounts as bulk chemicals in various industries, including polymers, fine chemicals, and commercial products.^39^ Benzoic acids^32,39,40^ and its analogues are biologically important scaffold as they are featured in many natural products and medically potent drugs (Fig. 1).^41–44^

They have a wide range of applications, including antimicrobial preservatives, antifungals, tablet and capsule lubricants, and UV protection agents.^45–47^ Utilizing a blend of medicinal plants that consist of Benzoic acid derivatives, amino acids, antioxidant chemicals, and certain minerals may be the optimal approach in formulating a phytomedicine for dealing with anemia or sickle cell disease (SCD).^43,48–50^ In 1971, Pierre and colleagues found that benzoic acid derivatives have anti-sickling properties *in vitro* using the root extract of *Fagara xanthoxyloides*.^48^ Similarly, Elekwa and his colleagues have revealed the anti-sickling benefits of *p*-fluorobenzoic acid.^51^

Hence, by deliberately avoiding hazardous substances, we have discovered that tosylhydrazones can undergo direct oxidative deprotection to produce benzoic acid derivatives (Scheme 1e). This developed method can be used in chemical industries as a cost-effective, efficient and ecologically benign synthesis.^52–58^ Herein, we report the metal and base-free deprotection of tosylhydrazones to the oxidative product of its parent aldehyde group using iodine and DMSO system with optimal conditions.

## Results and discussion

The investigation started by examining the reaction conditions using tosylhydrazone 1a as the model substrate.

Initially, reaction was conducted in the DMSO without the use of iodine at 100 °C for 1 h. Unfortunately, the reaction was failed to afford the target product 2a in the absence of iodine (Table 1, entry 1). Next, we attempted to manipulate the reaction environment by adding various reagents such as NaI, TBAI, NH_4_I and KI (Table 1, entries 2–5). However, using these reagents were futile and resulted in no product formation. Further, the use of iodine reagent in catalytic amount (10 mol% or 0.1 equiv.) in DMSO afforded the needed product 2a in 30% yield (Table 1, entry 6). It indicates that the iodine reagent is essential for this conversion. Subsequently, increasing the equivalent of iodine from 0.1 equiv. to 0.5, 1.0 and 1.5 equiv. improved the reaction efficiency to provide the 2a in 56%, 78% and 87% yields, respectively (Table 1, entries 7–9). Further, elevation in temperature from 100 to 120 °C also did not have any impactful effect on the productivity of this transformation (Table 1, entry 10). Reducing reaction temperature from 100 °C to room temperature and 70 °C significantly affected the reaction outcome, no reaction occurred at room temperature while only 60% yield of 2a was observed at 70 °C (Table 1, entries 11 and 12). Moreover, we noticed the reaction outcome by switching to the other solvents. Changing solvents from DMSO to toluene, acetonitrile (MeCN), 1,4-dioxane, water, MeOH, DMF, THF, chloroform (CHCl_3_) and DMA was entirely ineffective and 2a was either not observed or obtained in poor yields (Table 1, entries 13–22). It suggested that DMSO has a critical role in this reaction transformation. Finally, we found that the use of 1.5 equiv. of iodine in DMSO at 100 °C was the optimum condition for the satisfactory yield of 2a.

**Table 1:** Optimization of reaction conditions^a^

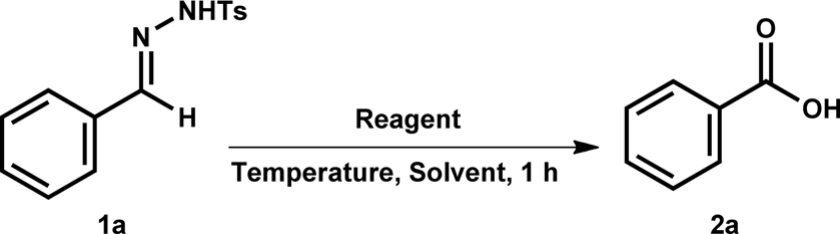				
Entry	Reagent (equiv.)	Solvent	*T* (°C)	Yield^b^ (%)
1	—	DMSO	100 °C	NR
2	NaI (1.0)	DMSO	100 °C	NR
3	TBAI (1.0)	DMSO	100 °C	NR
4	NH_4_I (1.0)	DMSO	100 °C	NR
5	KI (1.0)	DMSO	100 °C	NR
6	I_2_ (0.1)	DMSO	100 °C	30
7	I_2_ (0.5)	DMSO	100 °C	56
8	I_2_ (1)	DMSO	100 °C	78
**9**	**I**_**2**_ **(1.5)**	**DMSO**	**100 °C**	**87**
10	I_2_ (1.5)	DMSO	120 °C	86
11	I_2_ (1.5)	DMSO	RT	NR
12	I_2_ (1.5)	DMSO	70 °C	60
13	I_2_ (1.5)	Toluene	100 °C	15
14	I_2_ (1.5)	MeCN	80 °C	Trace
15	I_2_ (1.5)	1,4-Dioxane	100 °C	10
16	I_2_ (1.5)	H_2_O	100 °C	NR
17	I_2_ (1.5)	MeOH	65 °C	NR
18	I_2_ (1.5)	DMF	150 °C	NR
19	I_2_ (1.5)	THF	60 °C	NR
20	I_2_ (1.5)	CHCl_3_	60 °C	NR
21	I_2_ (1.5)	DMF	100 °C	NR
22	I_2_ (1.5)	DMA	100 °C	NR

a Reaction conditions: 1a (0.36 mmol), I_2_ (0.54 mmol), solvent (1.5 mL), 100 °C.

b Isolated yields. NR = no reaction. RT = room temperature.

Under optimized reaction conditions, the accessibility of the protocol was observed with the use of a range of benzaldehyde tosylhydrazones 1 (Table 2). The reaction of benzaldehyde tosylhydrazones with electron-donating substituents such as methyl (1b), methoxy (1c, 1f), and isopropyl (1e) either at *meta*- or *para*-position of aryl rings proceeded efficiently to deliver the corresponding products 2b (88%), 2c (83%), 2e (93%) and 2f (87%). It is worth mentioning that the oxidizable thiomethyl (1d) group could sustained in the optimized reaction condition to give 2d in a 96% yield. Tosylhydrazones of benzaldehyde having halogen substituents such as fluoro (1g) chloro (1h) and bromo (1i) also worked well, resulting in the production of the corresponding products 2g, 2h and 2i in high yields of 92%, 85% and 89%, respectively. The strong electron-withdrawing nitro group at the *para*-position of tosylhydrazone 1j had little impact on the oxidative deprotection process, giving 2j a reduced yield of 65%. It was interesting to observe that other electron-withdrawing groups *para*-cyano (1k), nitro (1l) and ester (–COOMe; 1l′) group at *meta*-position did not have any negative influence on reaction outcome, providing expected products 2k, 2l and 2l′ in excellent yields (87–93%). Furthermore, tosylhydrazones 1m and 1n with hydroxy substitutions reacted well to afford the 2m and 2n in good yields (73–88%). It is worth mentioning that tosylhydrazones with base-sensitive functional groups such as ester (1l′) and hydroxy (1m and 1n) groups were well tolerated under the optimized conditions. Delightfully, the reaction of 4-methyl-*N*′-(thiophen-2-ylmethylene)benzene-sulfonohydrazide (1o) showed credible reactivity to afford 2o in 93% yield.

**Table 2:** Oxidative deprotection of tosylhydrazones to benzoic acid derivatives^a^^,^^b^

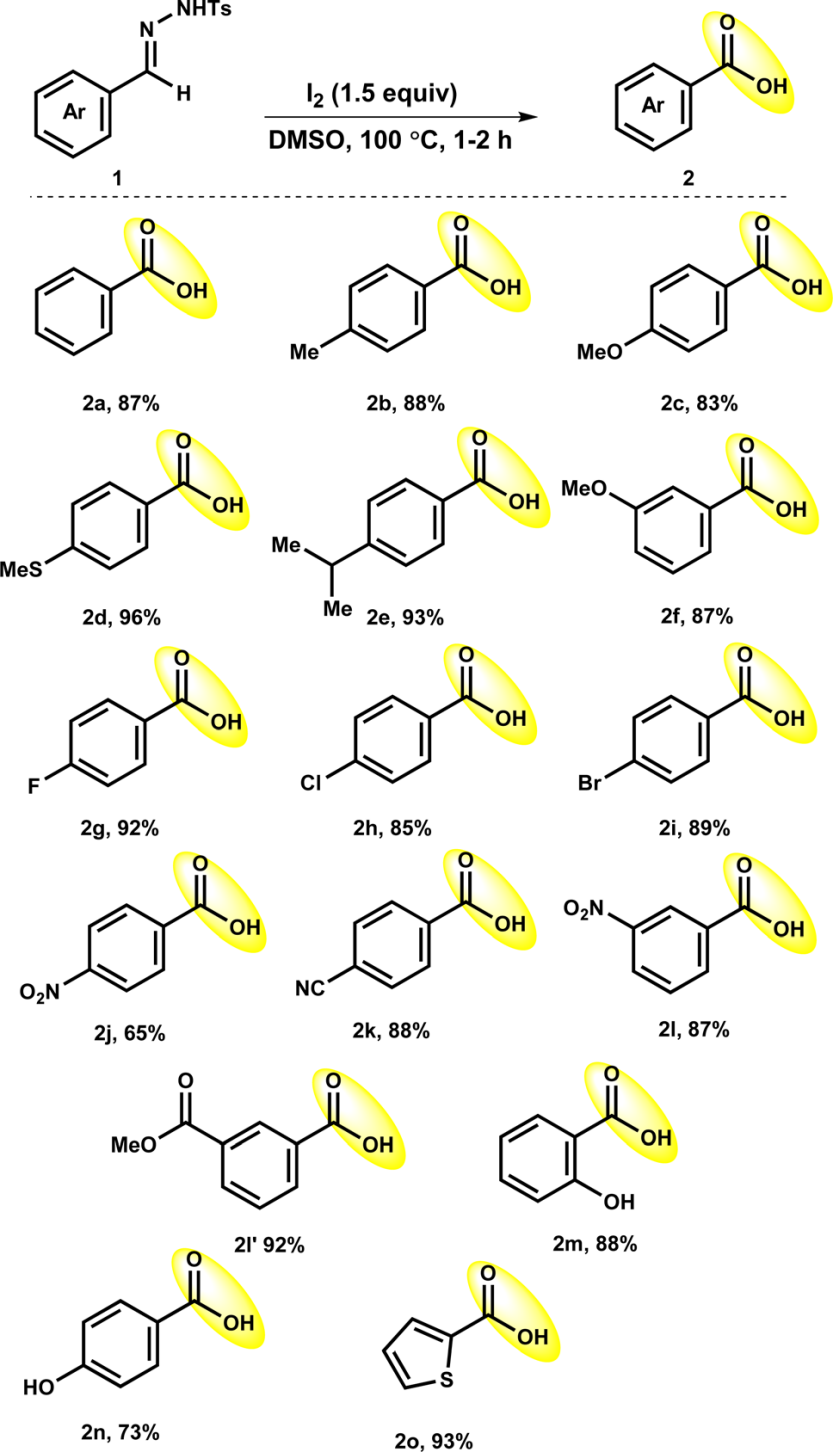

a Reaction conditions: 1a (0.36 mmol), I_2_ (0.54 mmol), solvent (1.5 mL), 100 °C.

b Isolated yields.

**Scheme 2:**
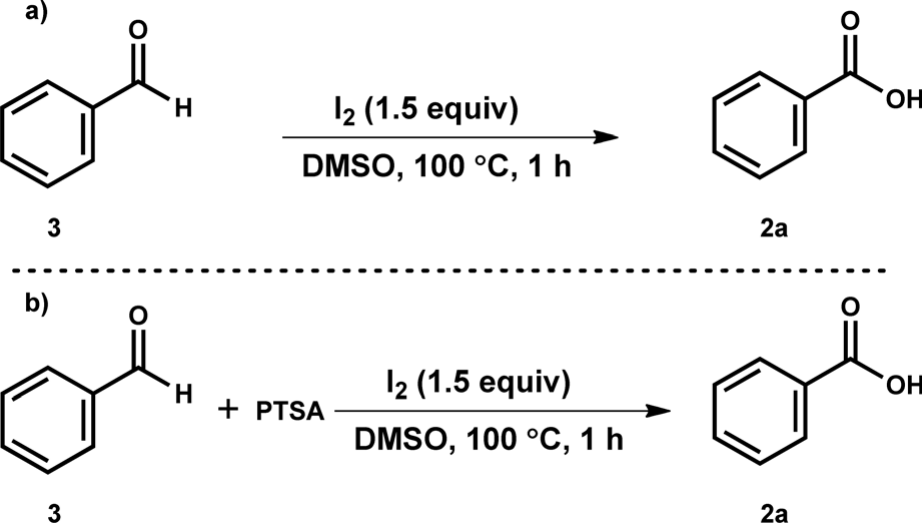
Control experiments.

Some of the control studies were designed to understand the mechanistic pathway (Scheme 2). Firstly, when the reaction of benzaldehyde 3 was performed under optimized reaction conditions, benzoic acid (2a) was observed only in trace amounts (Scheme 2a). Next, we speculated that *in situ* generated *p*-TSA acid might be involved as a catalyst in benzaldehyde to benzoic acid formation. Therefore, a reaction of 3 was performed with 0.5 equiv. of *p*-toluenesulfonic acid (*p*-TSA), but 2a formation was not observed. Further, we carried out the reaction mixture's LCMS analysis for the reaction of 1b under standard conditions. LCMS data suggested the generation of *p*-toluenesulfinic acid (TsH) instead of PTSA (see the ESI†). Hence, these experiments suggested no involvement of benzaldehyde intermediate in the developed oxidative deprotection process of *N*-tosylhydrazone.

**Scheme 3:**
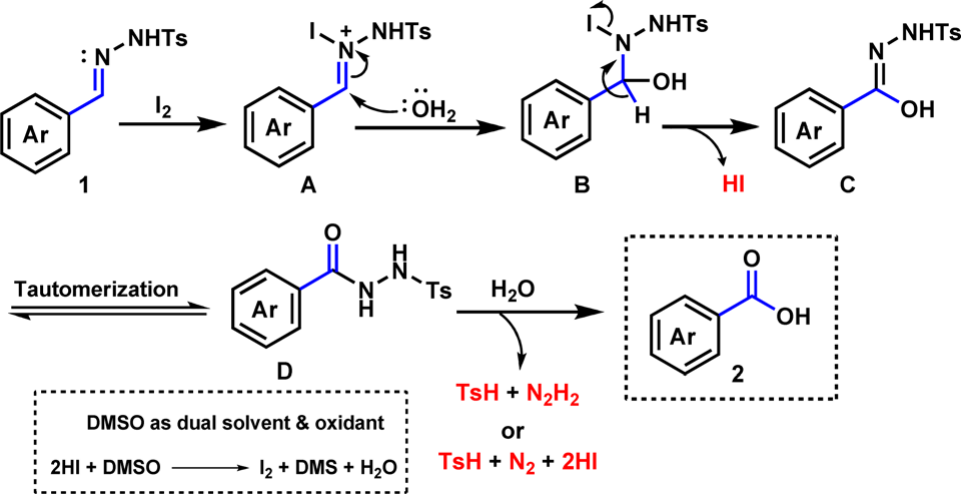
The plausible mechanism.

Based on experimental outcomes and previous studies,^59–63^ a plausible mechanism for the synthesis of benzoic acid is presented in Scheme 3. Firstly, *N*-tosylhydrazone is believed to form a nitrogen-iodine species A by electrophilic attack of iodine. Then nucleophilic attack of H_2_O molecule may lead to species B and further elimination of a HI molecule may afford species C. Tautomerization of intermediate C is likely to give species D. Finally, an attack of water molecule may lead to the formation of desired product 2 with the loss of *p*-toluenesulfinic acid (TsH) with diimide (N_2_H_2_) or TsH with nitrogen gas (N_2_) and hydrogen iodide (HI). The release of *p*-toluenesulfinic acid was confirmed by LCMS analysis (see the ESI†).

In conclusion, we have developed a feasible, economical, and environmentally benign method for the solvent-dependent oxidation of tosylhydrazone aldehydes into carboxylic acids. This particular transformation offers noteworthy benefits: (1) oxidation can be carried out without the need for an external catalyst, initiator, base, or additive; (2) the oxidation occurs under easy and mild reaction conditions, demonstrating excellent compatibility with functional groups, as shown by its ability to tolerate moisture, acid- and base-sensitive groups, as well as easily oxidizable groups. Considering these primary benefits, the current procedure can be considered as an important development in the field of oxidative deprotection of tosylhydrazones.

## Data availability

I have no conflict in data availability.

## Conflicts of interest

There are no conflicts to declare.

## Supplementary Material

RA-014-D4RA05849F-s001
