# Supplementary material for: Iodine/DMSO-catalyzed oxidative deprotection of N-tosylhydrazone for benzoic acid synthesis
Source: RSC Adv. 2024 Sep 24;14(41):30482–6. doi: 10.1039/d4ra05849f (PMC11421529; doi:10.1039/d4ra05849f)
Supplement: RA-014-D4RA05849F-s001 [file RA-014-D4RA05849F-s001.pdf]

Supplementary Information (SI) for RSC Advances.
This journal is © The Royal Society of Chemistry 2024

Supporting Information

"Iodine/DMSO-Catalyzed Oxidative Deprotection of *N*-Tosylhydrazone for Benzoic Acid Synthesis"

Rakshanda Singhal[a], Manish K. Mehra[b], Babita Malik[a], Meenakshi Pilania*[a]

[a*] *Department of Chemistry, Manipal University Jaipur, Jaipur (Rajasthan), VPO- Dehmi-Kalan, Off Jaipur-Ajmer Express Way, Jaipur (Rajasthan), India 303007*
meenakshi.pilania@jaipur.manipal.edu

[b*] *Department of Chemistry, Birla Institute of Technology and Science Pilani, Pilani Campus, Rajasthan, 333031 India. Current address: The Wistar Institute, Philadelphia, PA, 19104 USA*

Table of Contents

1. *General information* S2
2. *Experimental procedures* S2
3. *Characterization data for target compounds* S3 — S4
4. *Copies of ^{1}H and ^{13}C NMR spectra* S5 — S20
5. *LCMS Spectra of crude reaction mixture of* ***1b*** S21
6. *References* S21

1. General information

All substrates and reagents were commercially available and used without further purification. All reagents were weighed and handled in air at room temperature. ^{1}H spectra were recorded in $CDCl_3$ or DMSO-d_6 on 400 MHz NMR spectrometers and resonances (δ) are given in parts per million (ppm) relative to tetramethylsilane. Data are reported as follows: chemical shift, multiplicity (s = singlet, d = doublet, t = triplet, m = multiplet), coupling constants (Hz) and integration. ^{13}C spectra were recorded in $CDCl_3$ or DMSO-d_6 on 100 MHz NMR spectrometers and resonances (δ) are given in ppm. High-resolution mass spectra (HRMS) further measured for some of the compounds. Melting points were determined using lab melting point apparatus.

2. Experimental procedure

2.1 General procedure for the synthesis of *N*-tosylhydrazones (1)

A mixture of aryl or heteroaryl aldehydes (9.42 mmol) and tosylhydrazine (9.42 mmol) was added to a round bottomed flask in methanol (8 mL). The mixture was heated at 60 °C for 0.5 to 3 h to obtain the corresponding *N*-tosylhydrazones as white precipitate, which was filtered off and washed with diethyl ether (5 mL × 3) and dried under vacuum to obtain pure compound.

2.2 General procedure for the synthesis of benzoic acid (2)

A mixture of *N*-tosylhydrazone **1** (0.364 mmol) and iodine (0.546 mmol) in DMSO (1.5 mL) was stirred at 100℃ for 1 h. After completion of the reaction (monitored by TLC), the reaction mixture was cooled to room temperature. The mixture was then quenched with an aqueous saturated solution of $Na_2S_2O_3$ (5 mL) and organic phase was extracted with EtOAc (3 × 10 mL). The combined organic layers were washed with brine, dried over anhydrous Na_2SO_4, and concentrated under reduced pressure to get a solid compound. Obtained solid was washed with hexane and dried under high vacuum to the get pure desired product **2** in 85-95% yield.

3. Characterization data for target compounds

Benzoic acid (2a)[1-3]: Yield 87%, white solid, melting point 120-122°C, ^{1}H NMR (400 MHz, $CDCl_3$) δ 8.10 – 8.00 (m, 2H), 7.59 – 7.51 (m, 1H), 7.42 (t, *J* = 7.7 Hz, 2H); ^{13}C NMR (101 MHz, $CDCl_3$) δ 172.03, 133.83, 130.23, 129.30, 128.51.

4-Methylbenzoic acid (2b) [1-3]: Yield 88%, yellowish solid, melting point 181-182°C, ^{1}H NMR (400 MHz, DMSO-d_6) δ 12.79 (s, 1H), 7.85 (d, *J* = 7.9 Hz, 2H), 7.29 (d, *J* = 7.9 Hz, 2H), 2.36 (s, 3H); ^{13}C NMR (100 MHz, DMSO-d_6) δ 167.81, 143.46, 129.69 (d, *J* = 23.5 Hz), 21.57.

4-Methoxybenzoic acid (2c)[1-3]: Yield 83%, yellowish solid, melting point 183-184°C, ^{1}H NMR (400 MHz, DMSO-d_6) δ 12.63 (s, 1 H), 7.89 (d, *J*= 8.8 Hz, 2 H), 7.02 (d, *J*= 8.8 Hz, 2 H), 3.82 (s, 3 H); ^{13}C NMR (100 MHz, DMS-d_6) δ 167.5, 163.3, 131.8, 123.5, 114.3, 55.9.

4-(Methylthio)benzoic acid (2d) [1-3]: Yield 96%, white solid, melting point 191-195°C, ^{1}H NMR (400 MHz, DMSO-d_6) δ 12.84 (s, 1H), 7.86 (d, *J* = 8.1 Hz, 2H), 7.32 (d, *J* = 8.2 Hz, 2H), 2.55 (s, 3H); ^{13}C NMR (100 MHz, DMSO-d_6) δ 167.53, 145.25, 130.18, 127.19, 125.30, 14.41.

4-Isopropylbenzoic acid (2e))[1-3]: ^{1}H NMR (400 MHz, DMSO- d_6) δ 12.81 (s, 1 H), 7.86 (d, *J* = 8.4 Hz, 2 H), 7.36 (d, *J* = 8.0 Hz, 2 H), 3.01 - 2.90 (m, 1 H), 1.21 (d, *J* = 6.8 Hz, 6 H); ^{13}C NMR (100 MHz, DMSO-d_6) δ 167.8, 154.1, 130.0, 128.9, 127.0, 34.0, 24.1.

3-Methoxybenzoic acid (2f) [1-3]: Yield 87%; white solid; melting point 103-108°C, ^{1}H NMR (400 MHz, $CDCl_3$) δ 7.73 (dt, *J* = 7.6, 1.3 Hz, 1H), 7.63 (dd, *J* = 2.7, 1.5 Hz, 1H), 7.39 (t, *J* = 7.9 Hz, 1H), 7.16 (ddd, *J* = 8.3, 2.7, 1.0 Hz, 1H), 3.87 (s, 3H); ^{13}C NMR (100 MHz, $CDCl_3$) δ 171.94, 159.62, 130.62, 129.55, 122.70, 120.48, 114.39, 55.48.

4-Fluorobenzoic acid (2g) [1-3]: Yield 92%, white solid; melting point 182-186°C, ^{1}H NMR (400 MHz, DMSO-d_6) δ 7.95 – 7.88 (m, 2H), 7.47 (dd, *J* = 8.2, 6.6 Hz, 2H); ^{13}C NMR (100 MHz, DMSO) δ 166.9, 164.2, 132.7, 127.9, 116.2.

4-Chlorobenzoic acid (2h) [1-3]: Yield 85%, white solid, melting point 234-242°C , ^{1}H NMR (400 MHz, DMSO-d_6) δ 8.10 – 7.90 (m, 2H), 7.55 – 7.34 (m, 2H); ^{13}C NMR (100 MHz, DMSO-d_6) δ 167.93, 139.24, 131.17, 128.97, 128.56.

4-Bromobenzoic acid (2i)[1-3]: Yield 89%, white solid, melting point 251-252°C, ^{1}H NMR (400 MHz, DMSO-d_6) δ 13.19 (s, 1H), 7.86 (d, *J* = 8.1 Hz, 2H), 7.70 (d, *J* = 8.1 Hz, 2H); ^{13}C NMR (100 MHz, DMSO-d_6) δ 167.1, 132.14, 131.75, 130.47, 127.34.

4-Nitrobenzoic acid (2j) [1-3]: Yield 65%, white solid, melting point 236-238°C, ^{1}H NMR (400 MHz, DMSO-d_6) δ 13.65 (s, 1H), 8.34 – 8.25 (m, , *J* = 8.8 Hz, 2H), 8.20 – 8.10 (m, *J* = 8.8 Hz, 2H); ^{13}C NMR (100 MHz, DMSO-d_6) δ 166.25, 150.45, 136.84, 131.13, 124.13.

4-Cyanobenzoic acid (2k) [1-3]: Yield 88%, white solid, melting point 214-220°C, ^{1}H NMR (400 MHz, DMSO-d_6) δ 13.58 (s, 1H), 8.11 – 8.05 (m, 2H), 8.02 – 7.95 (m, 2H); ^{13}C NMR (100 MHz, DMSO-d_6) δ 166.53, 135.32, 133.15, 130.39, 118.66, 115.53.

3-Nitrobenzoic acid (2l) [1-3]: Yield 87%, yellowish solid, melting point 140-142°C; ^{1}H NMR (400 MHz, $CDCl_3$) δ 8.87 (t, *J* = 2.0 Hz, 1H), 8.43 – 8.33 (m, 2H), 7.63 (t, *J* = 8.0 Hz, 1H); ^{13}C NMR (100 MHz, $CDCl_3$) δ 167.58, 148.34, 135.71, 131.56, 125.10.

3-(Methoxycarbonyl)benzoic acid (2l'): Yield 92%, ^{1}H NMR (400 MHz, DMSO-d^6) δ 13.31 (s, 1H), 8.47 (t, *J* = 1.6 Hz, 1H), 8.21 – 8.14 (m, 2H), 7.65 (t, *J* = 7.8 Hz, 1H), 3.88 (s, 3H); ^{13}C NMR (100 MHz, DMSO- d_6) δ 166.9, 166.0, 134.2, 133.6, 131.8, 130.5, 130.2, 129.7, 52.8.

2-Hydroxybenzoic acid (2m) [1-3]: Yield 88%; yellowish solid; melting point 158-160 °C; ^{1}H NMR (400 MHz, DMSO-d_6) δ 11.32 (bs, 2H), 7.79 (dd, *J* = 7,9 Hz, 1.8 Hz 1H), 7.51 (distorted td, 1H), 6.96 – 6.90 (m, 2H); ^{13}C NMR (100 MHz, DMSO-d_6) δ 172.1, 161.3, 135.8, 130.4, 119.3, 117.2, 112.9.

4-Hydroxybenzoic acid (2n) [1-3]: Yield 73%, yellowish solid, melting point 213-217°C , ^{1}H NMR (400 MHz, DMSO-d_6) δ 12.42 (s, 1 H), 10.21 (s, 1 H), 7.78 (d, *J* = 8.8 Hz, 2 H), 6.82 (d, *J* = 8.4 Hz, 2H); ^{13}C NMR (100 MHz, DMSO-d_6) δ 167.7, 162.1, 132.0, 121.8, 115.6.

Thiophene-2-carboxylic acid (2o) [1-3]: Yield 93%, yellowish solid; melting point 135-145°C, ^{1}H NMR (400 MHz, DMSO- d_6) δ 13.07 (s, 1H), 7.89-7.73 (dd, 1H), 7.73 (dd, 1 H), 7.18 (dd, 1H); ^{13}C NMR (100 MHz, DMSO- d_6) δ 163.4, 135.1, 133.8, 133.7, 128.7.

Benzoic acid(2a)

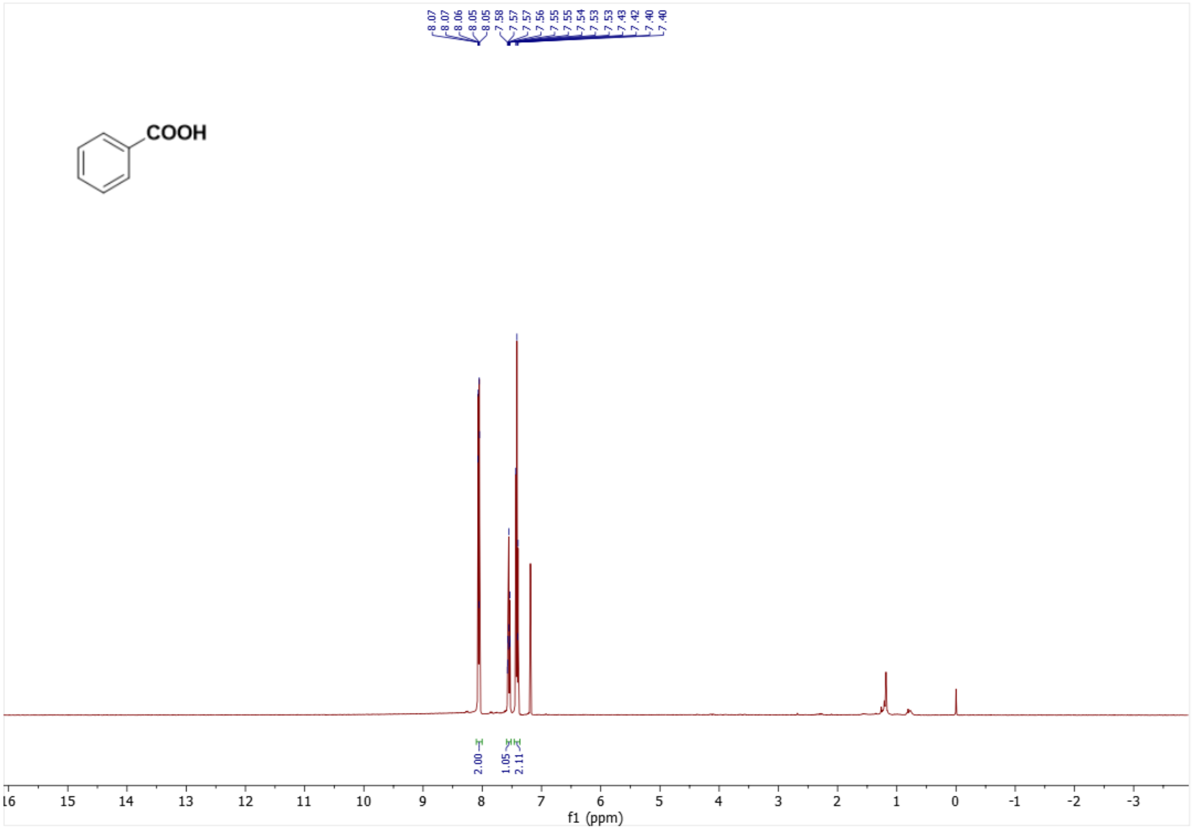

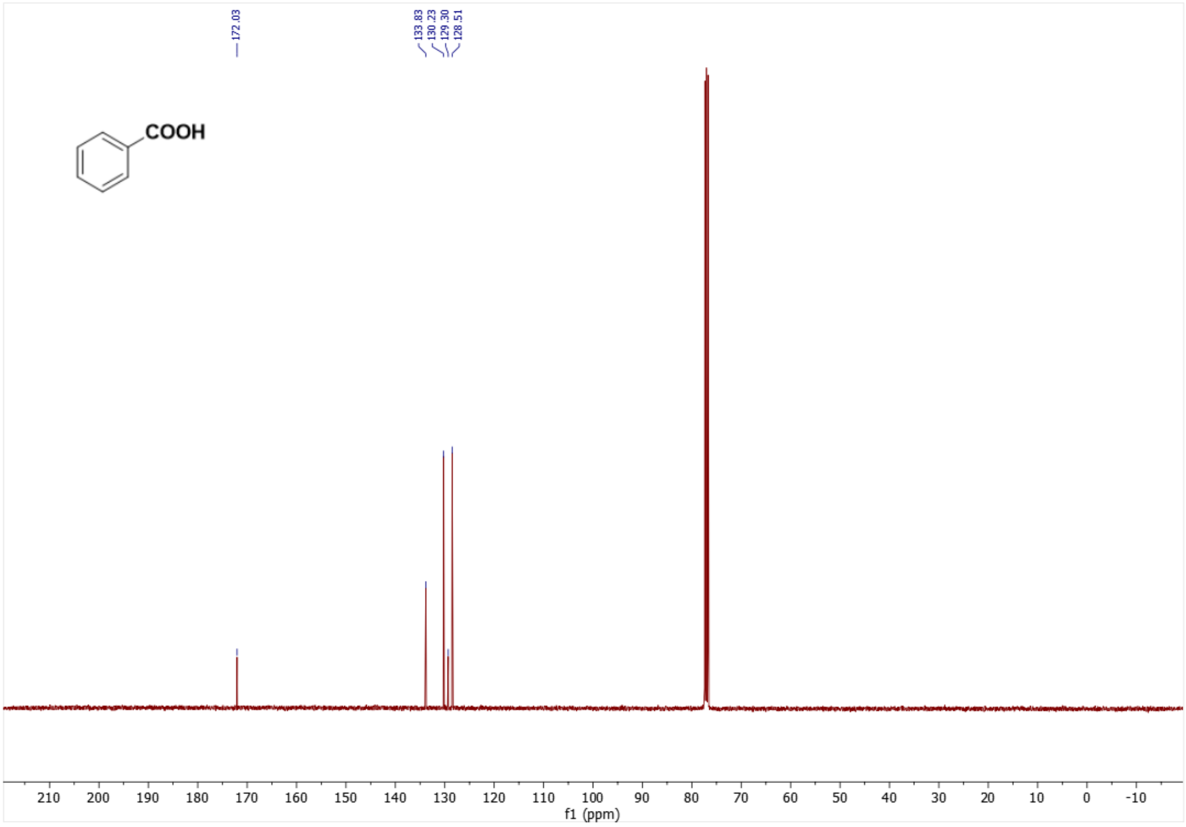

4-Methylbenzoic acid (2b)

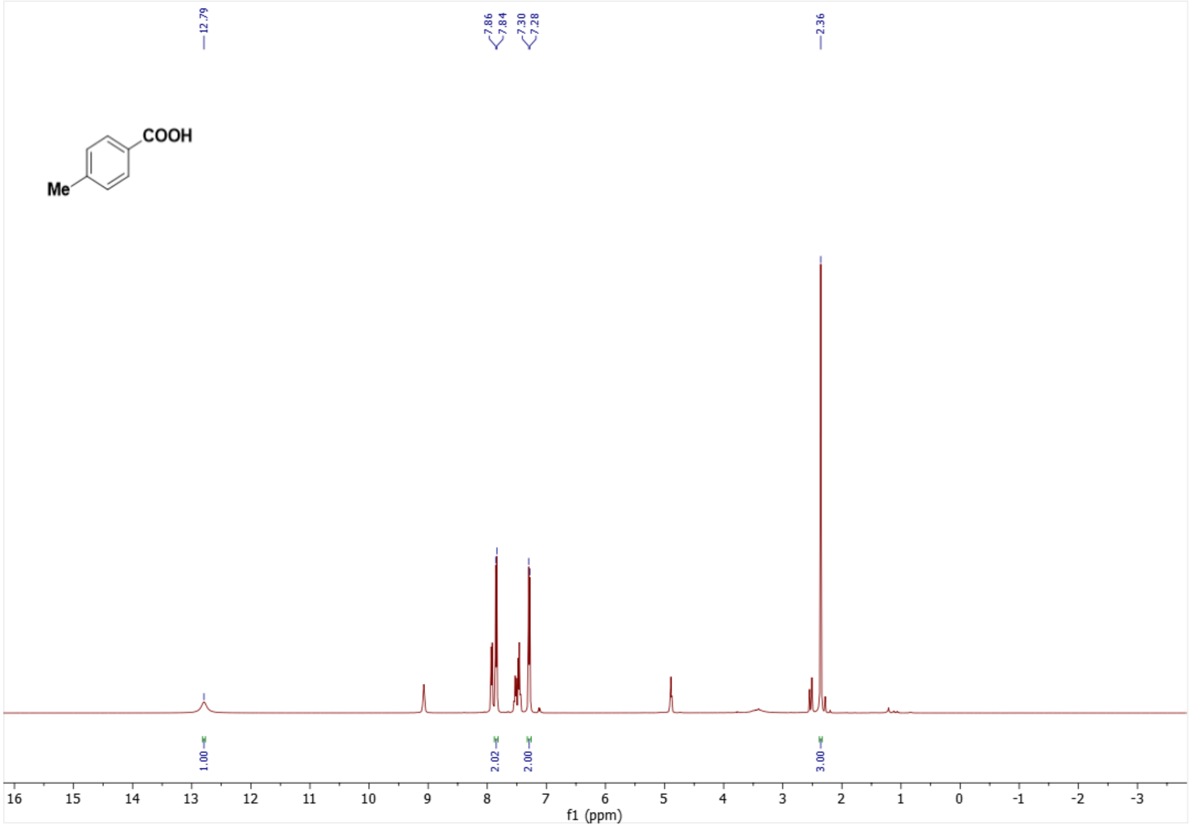

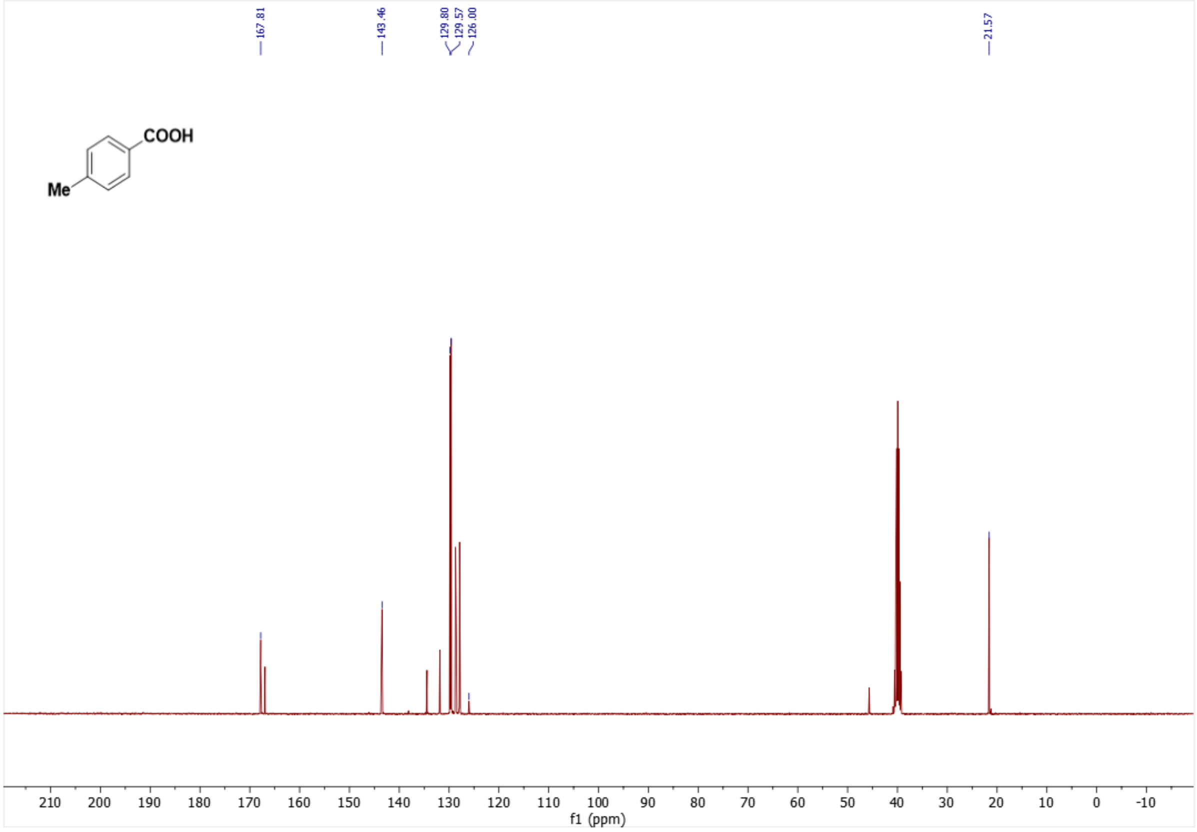

4-Methoxybenzoic acid (2c)

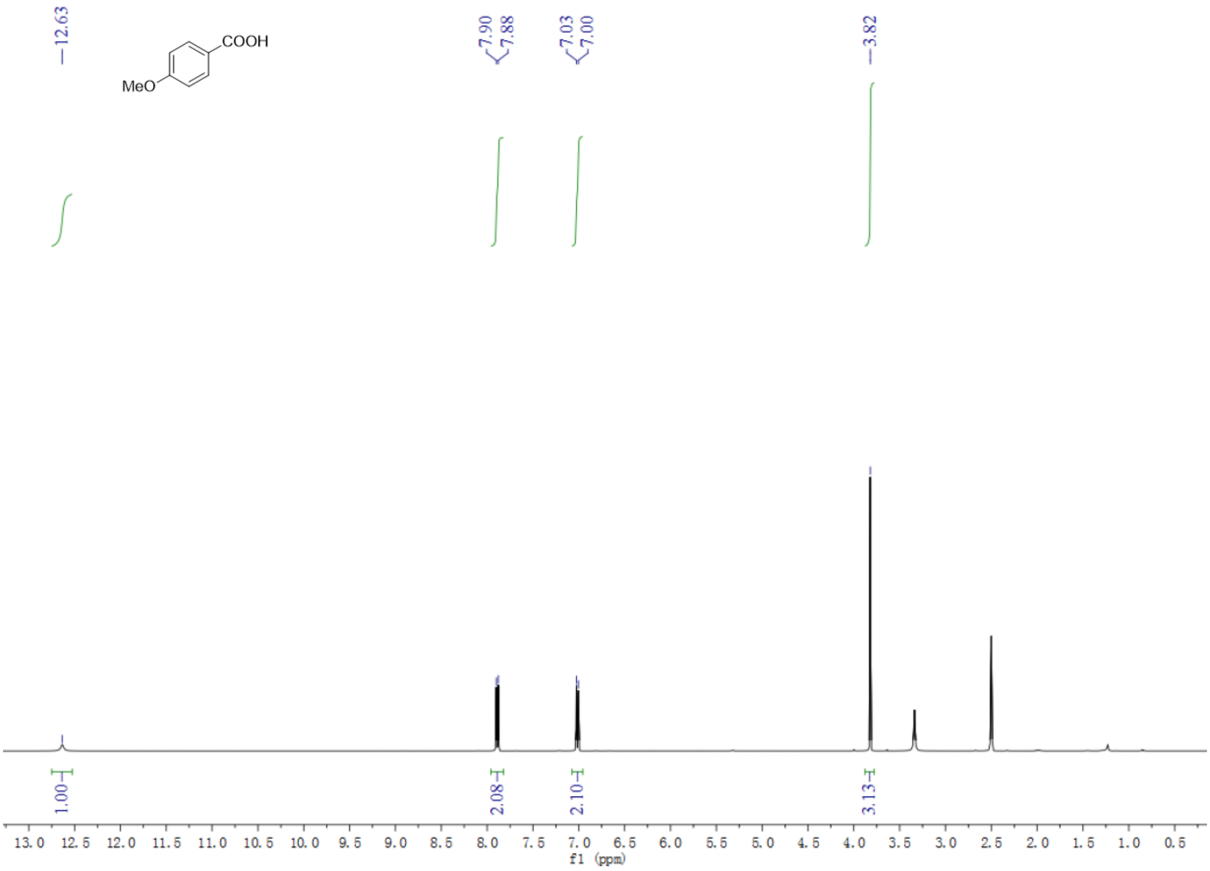

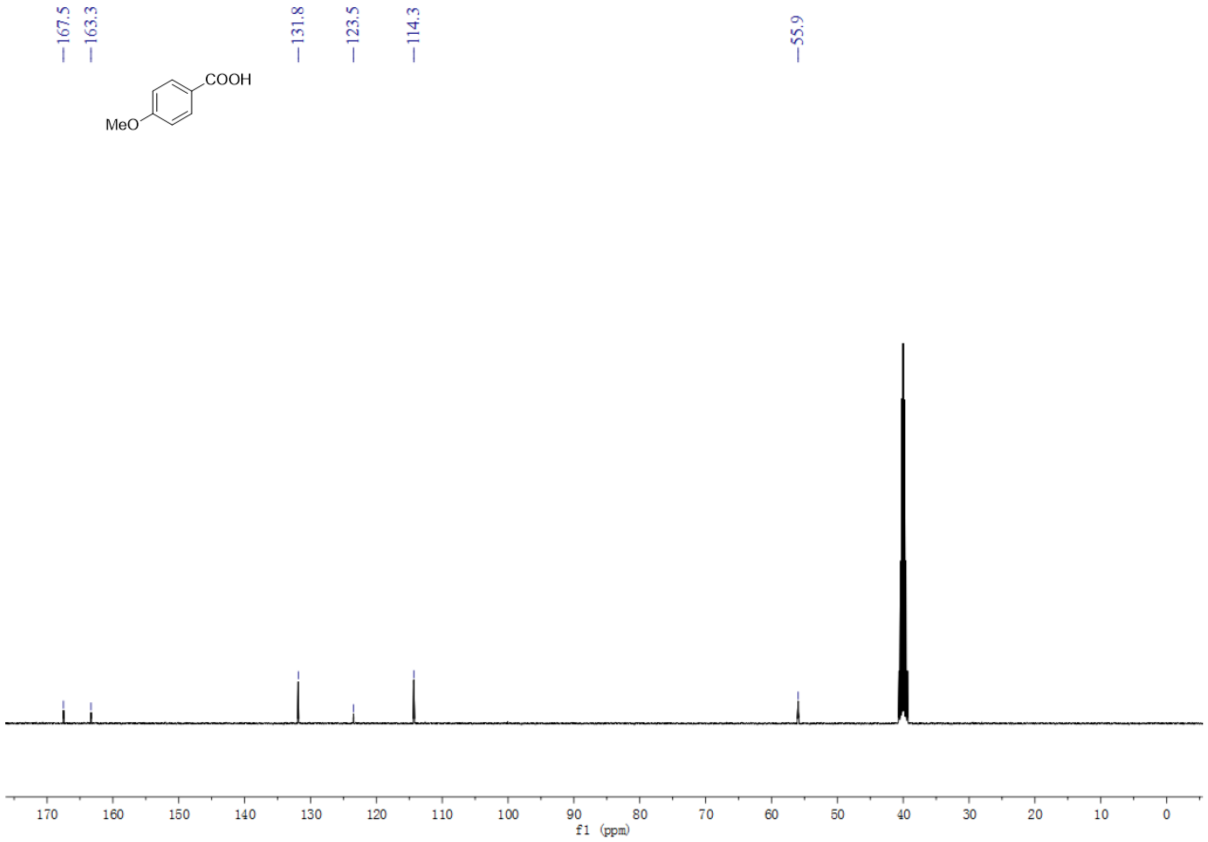

4-(Methylthio)benzoic acid (2d)

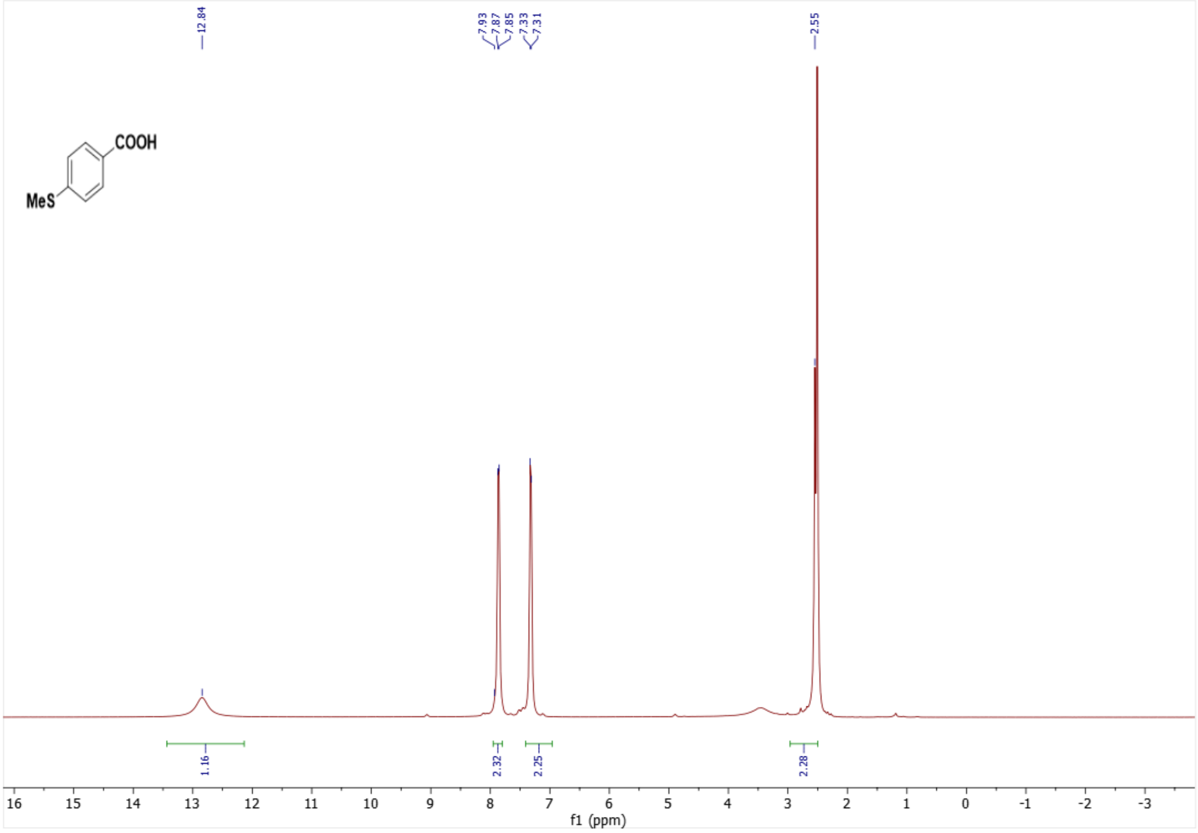

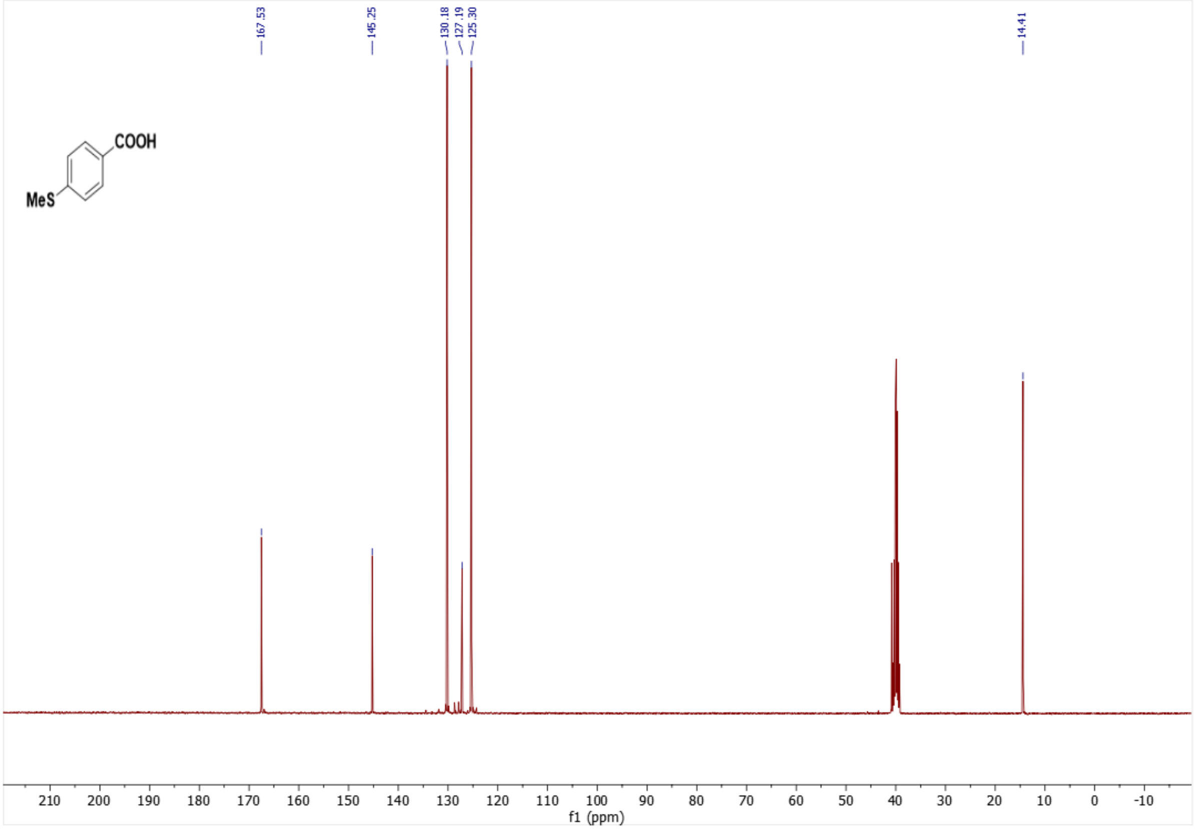

4-Isopropylbenzoic acid (2e)

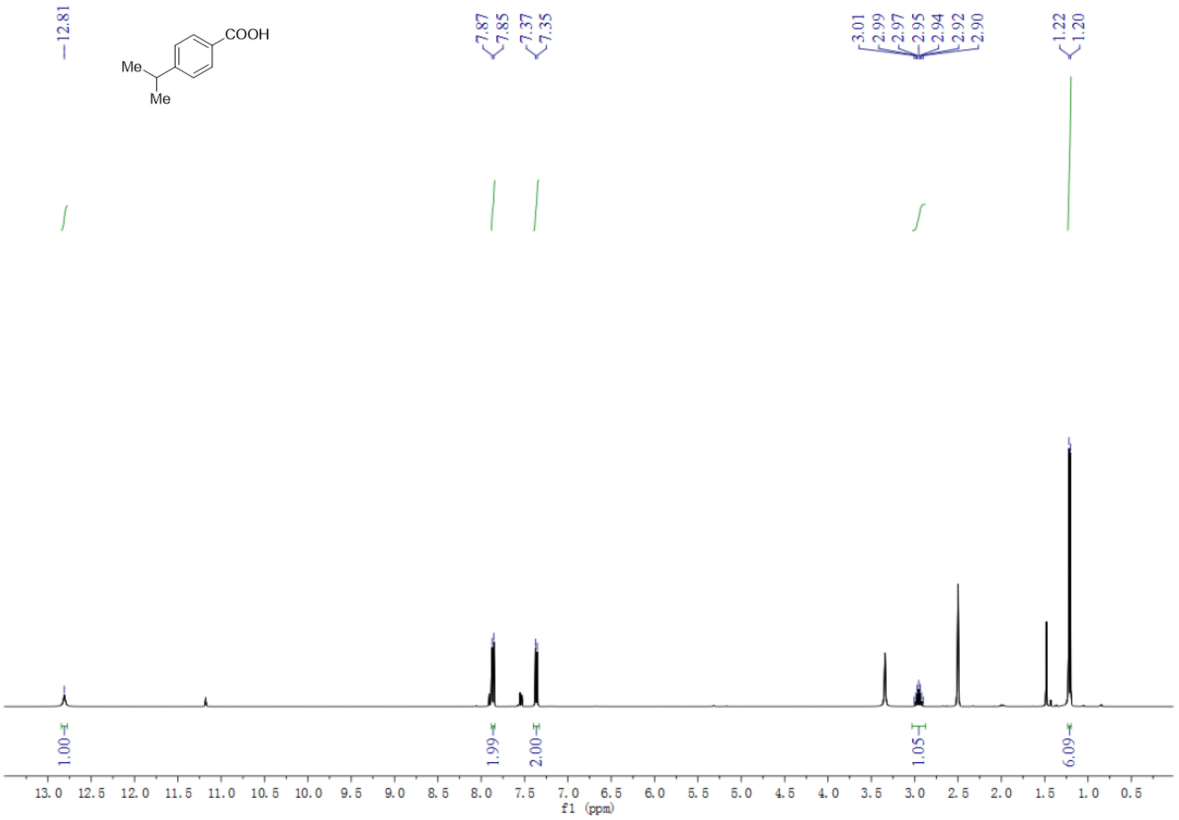

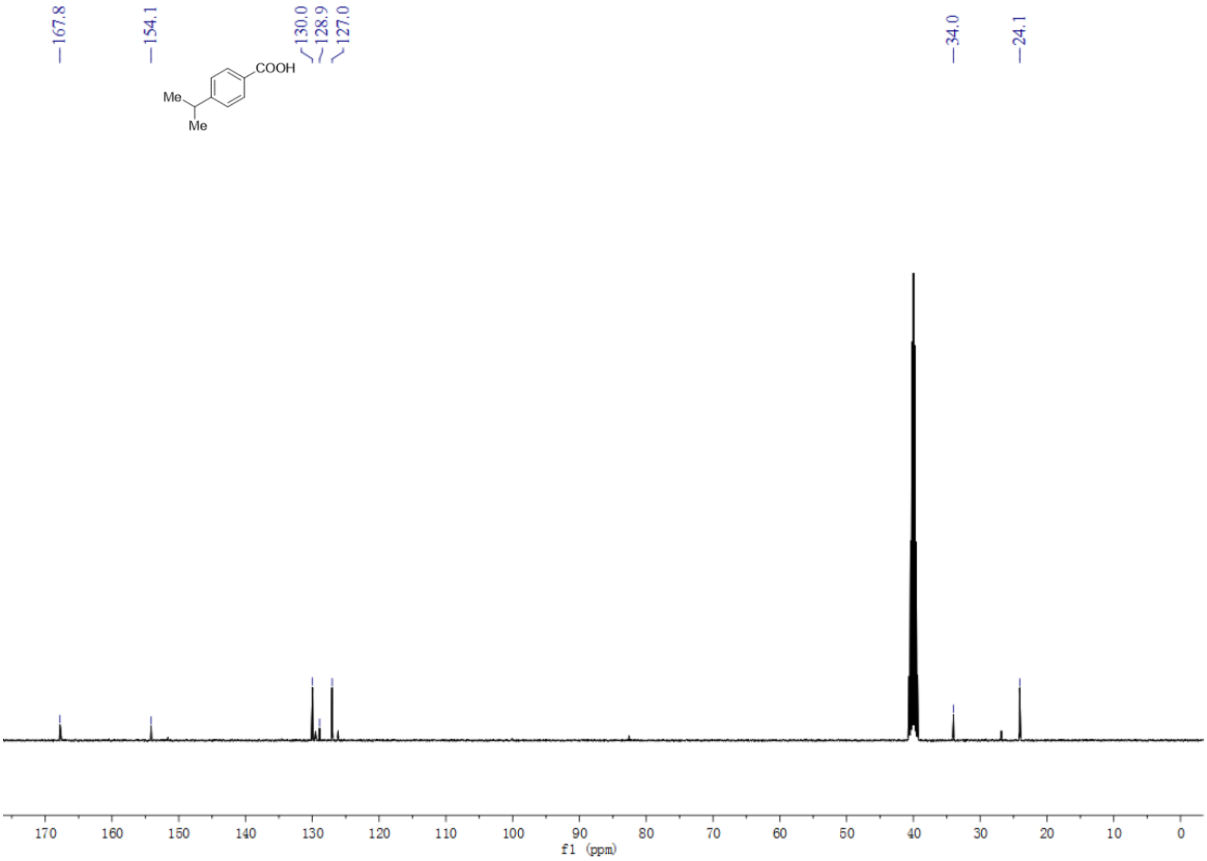

3-Methoxy benzoic acid (2f)

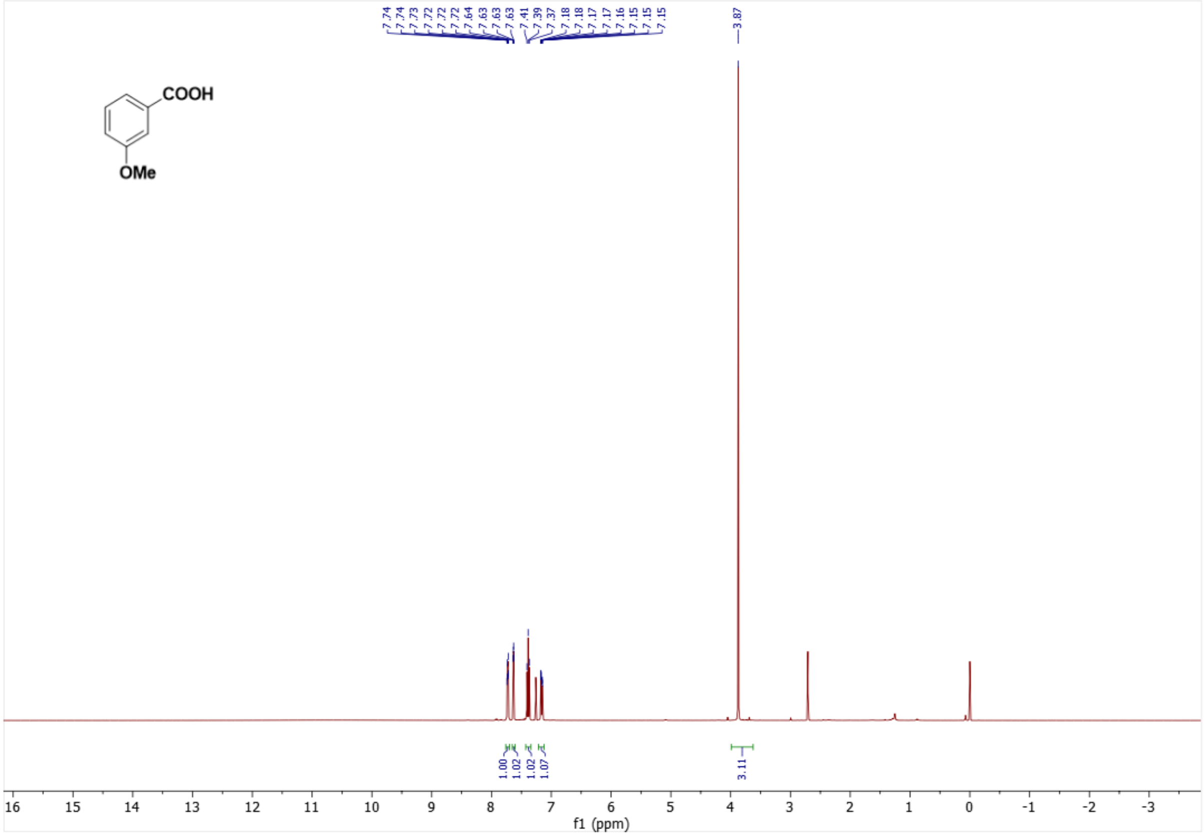

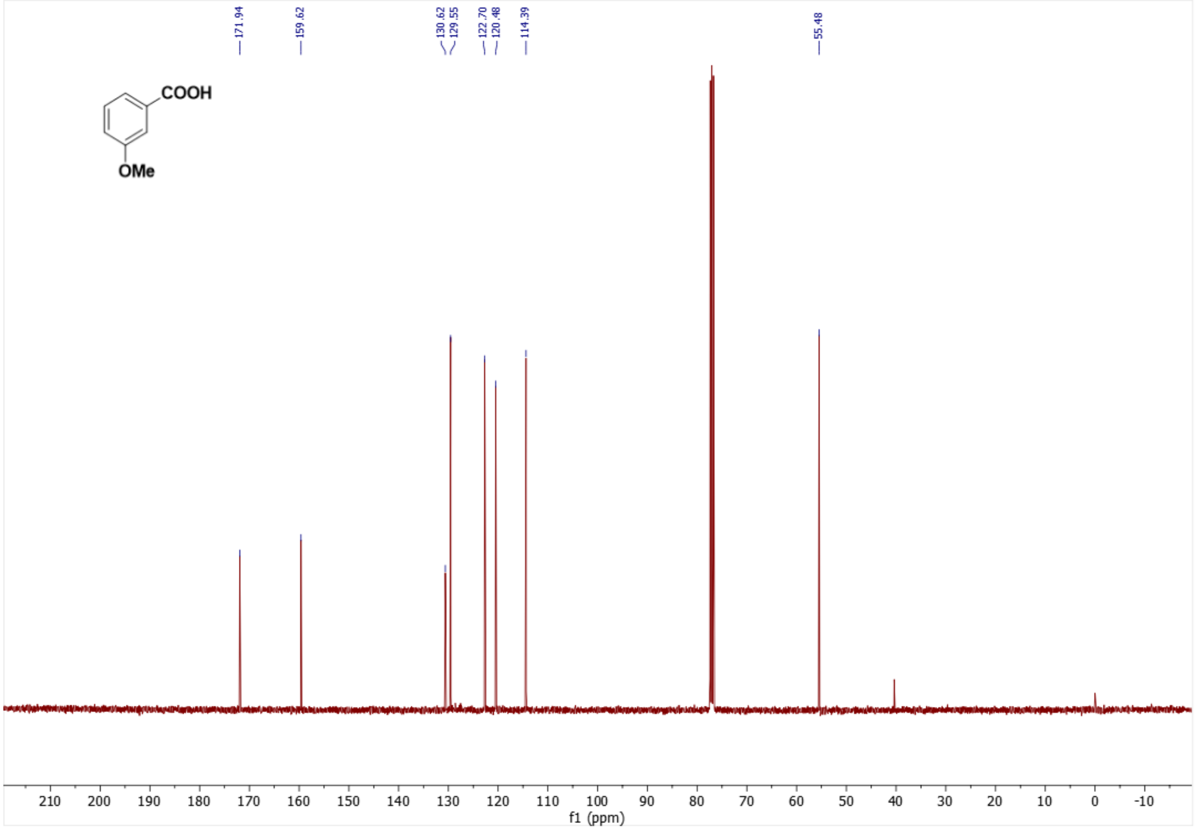

4-Fluorobenzoic acid (2g)

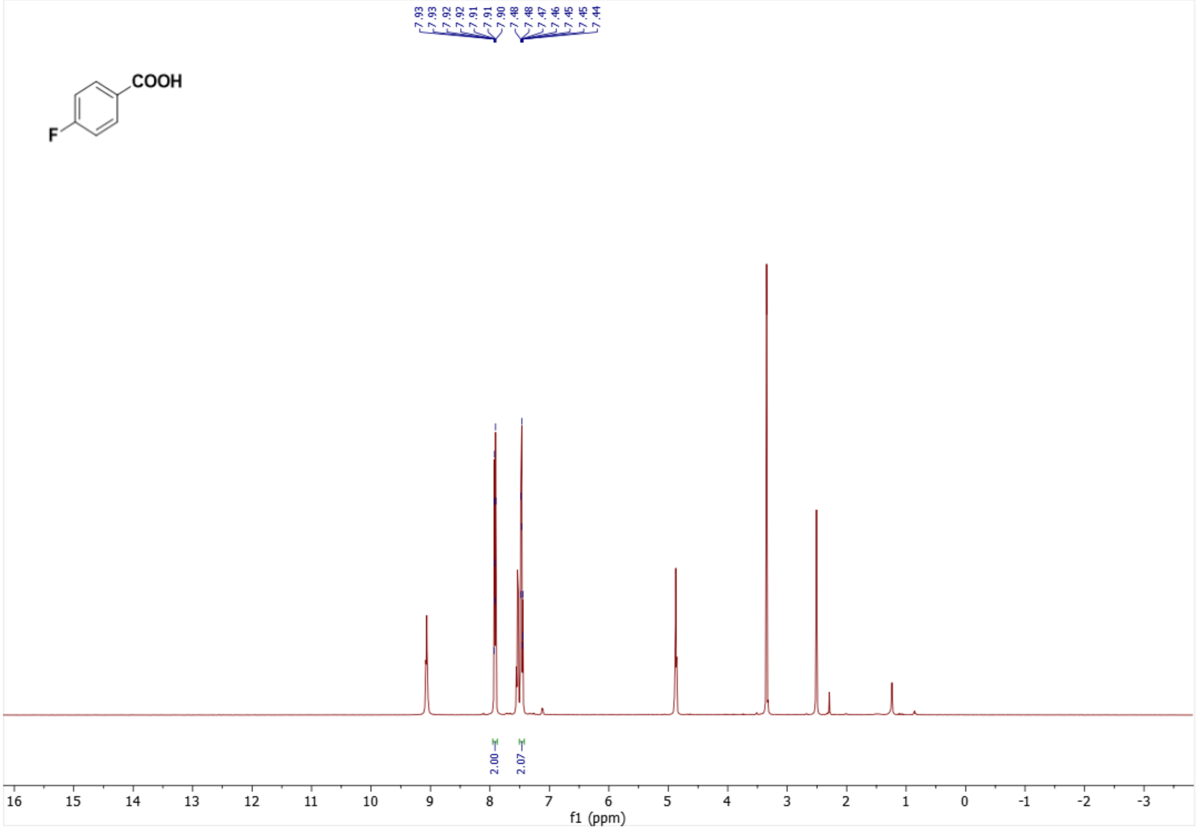

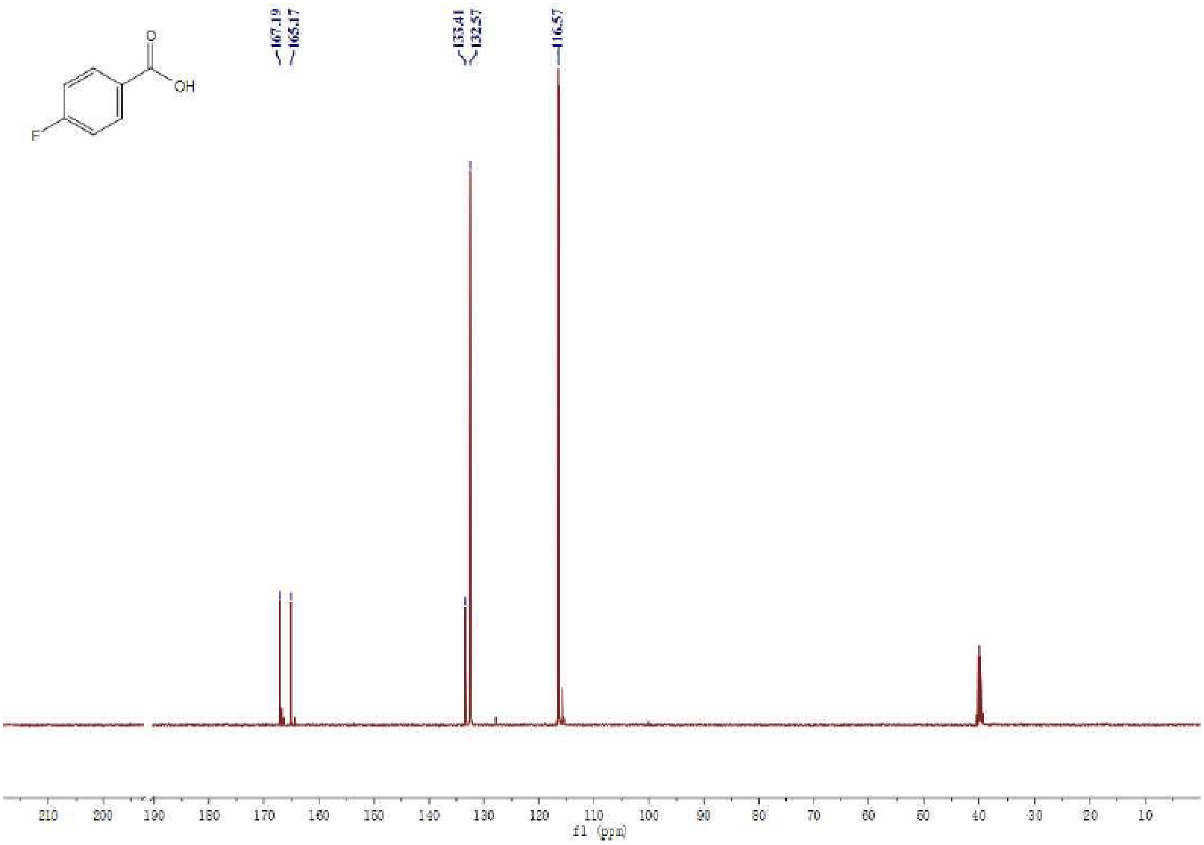

4-Chlorobenzoic acid (2h)

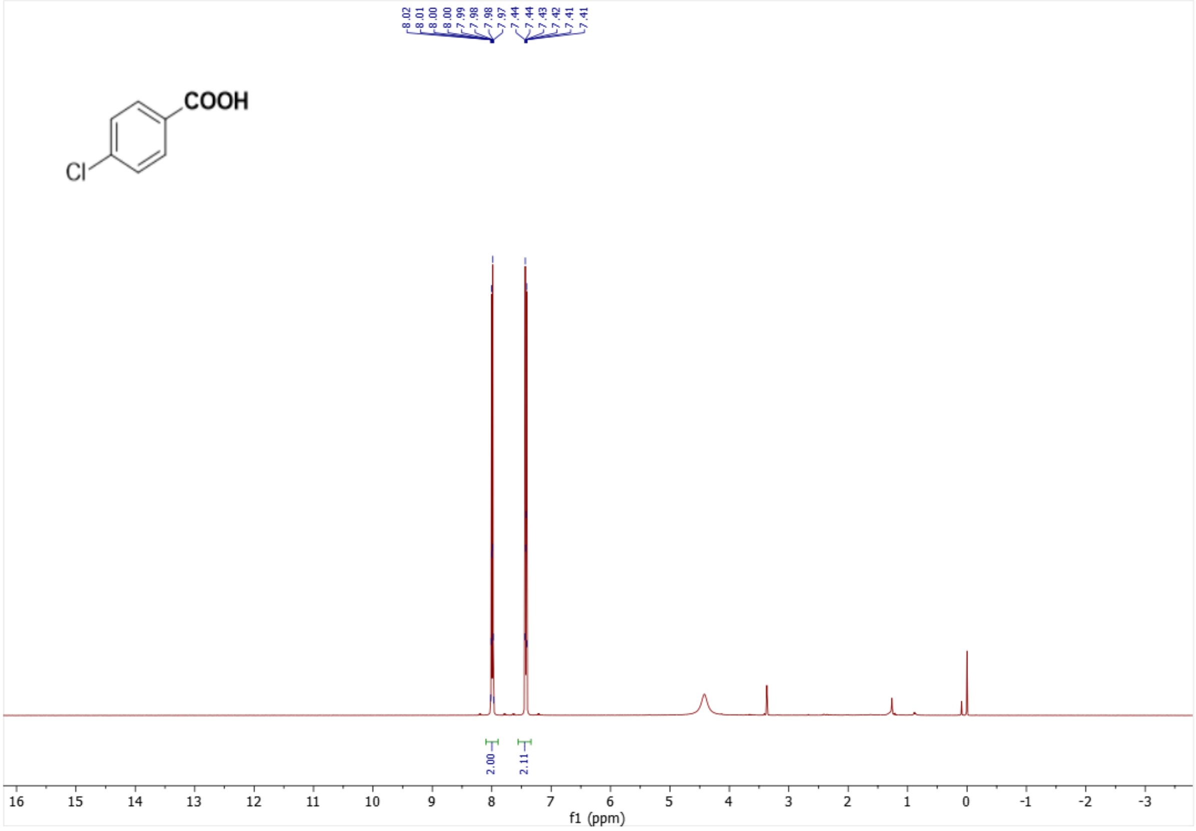

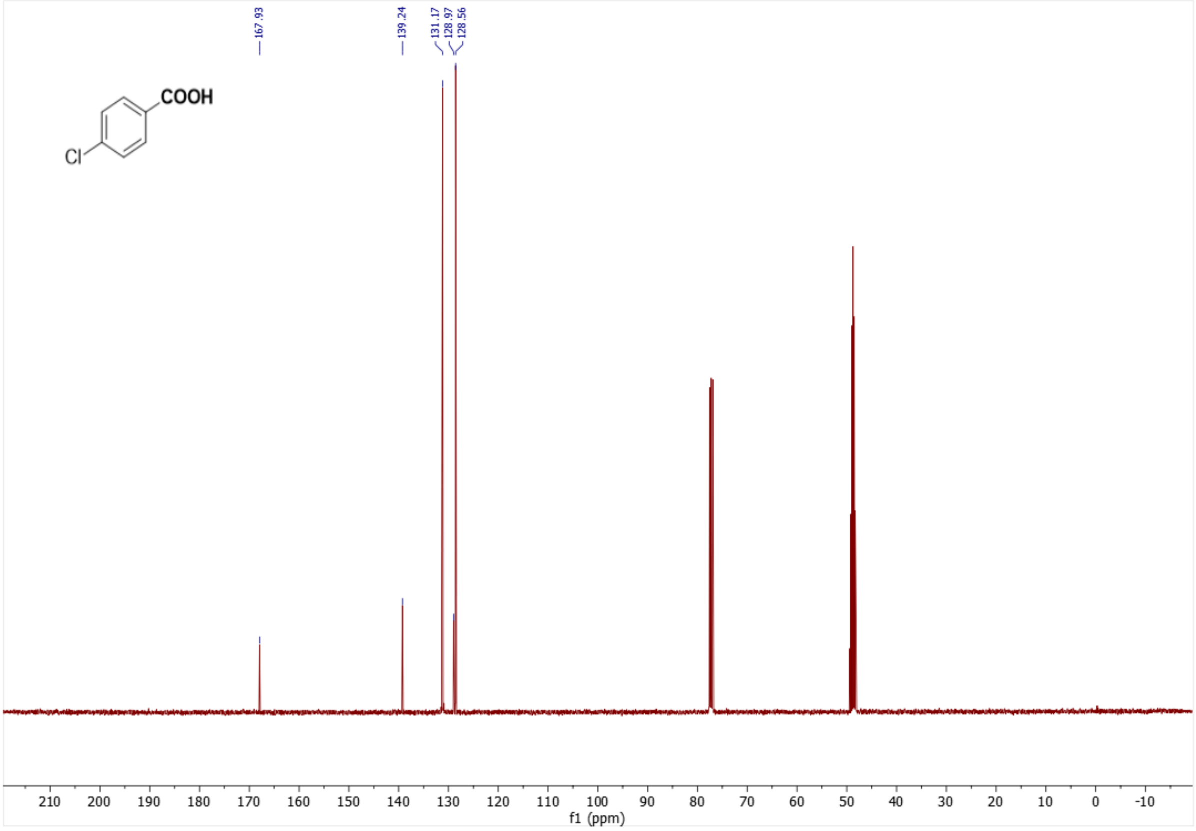

4-Bromobenzoic acid (2i)

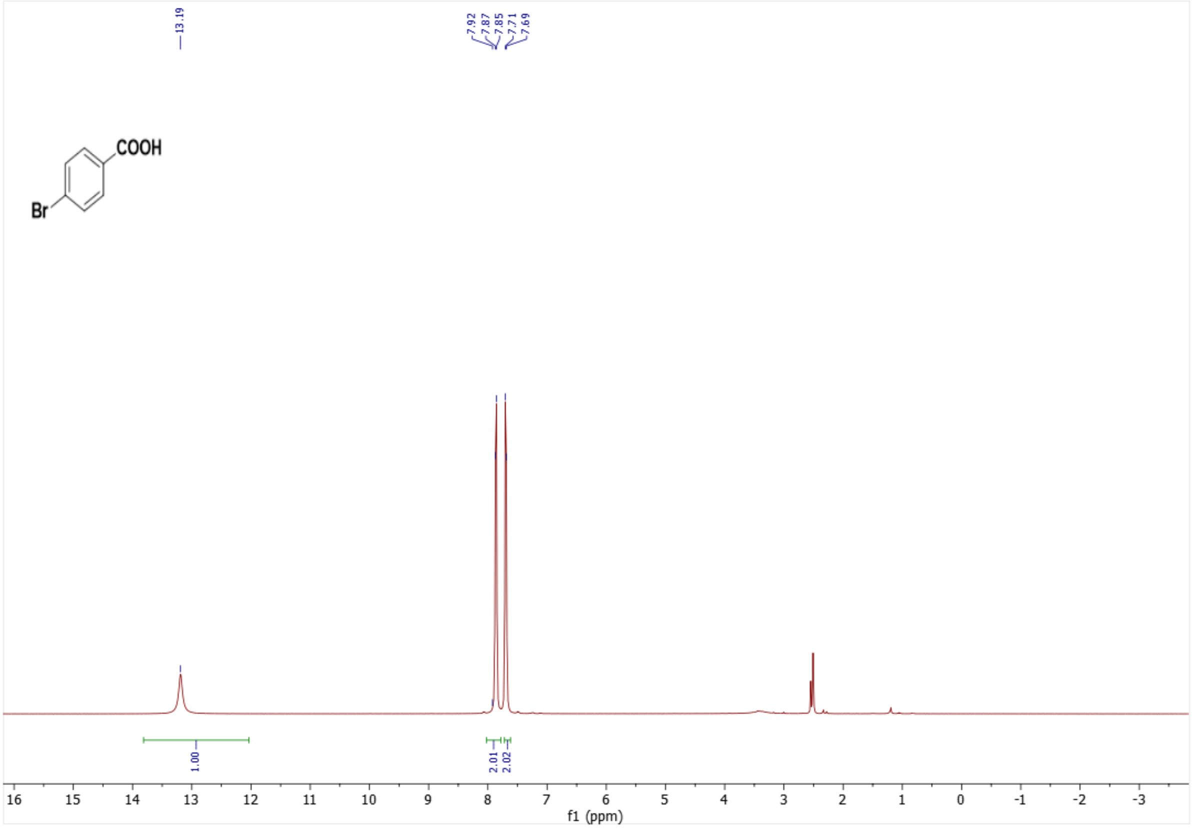

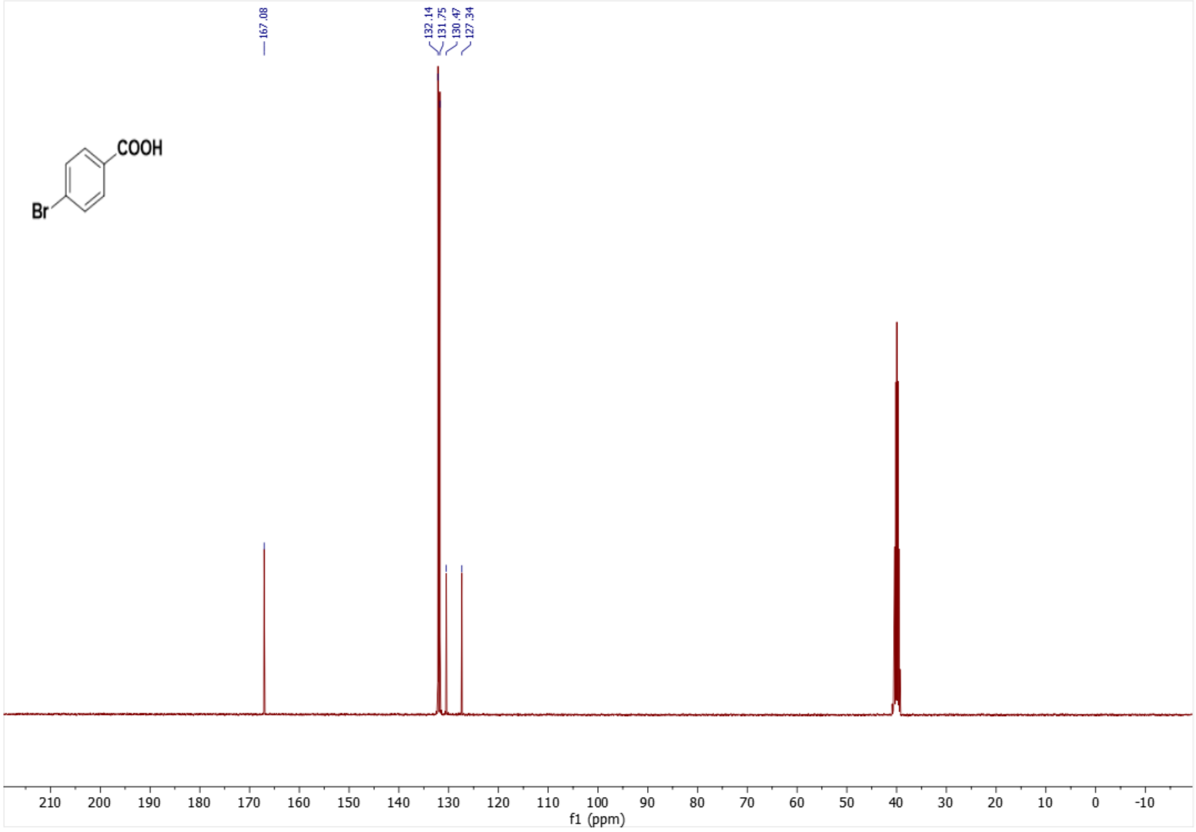

4-Nitro benzoic acid (2j)

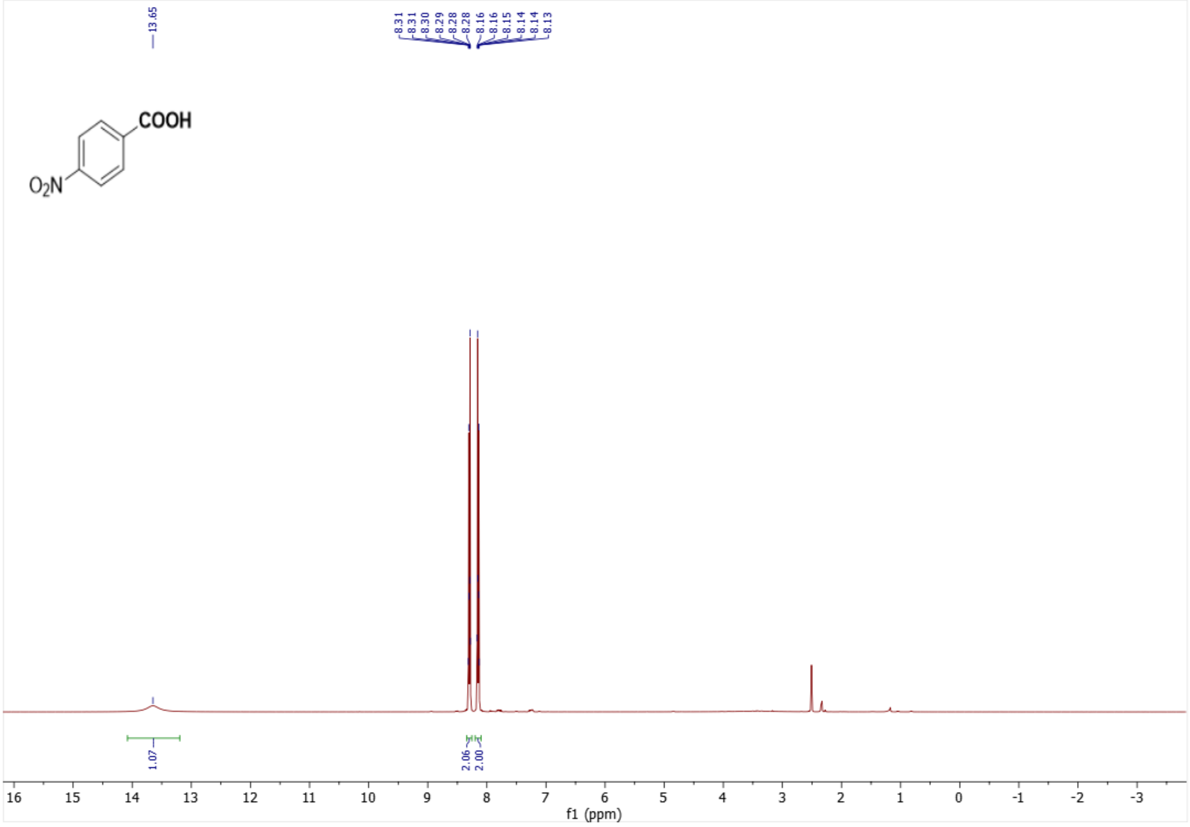

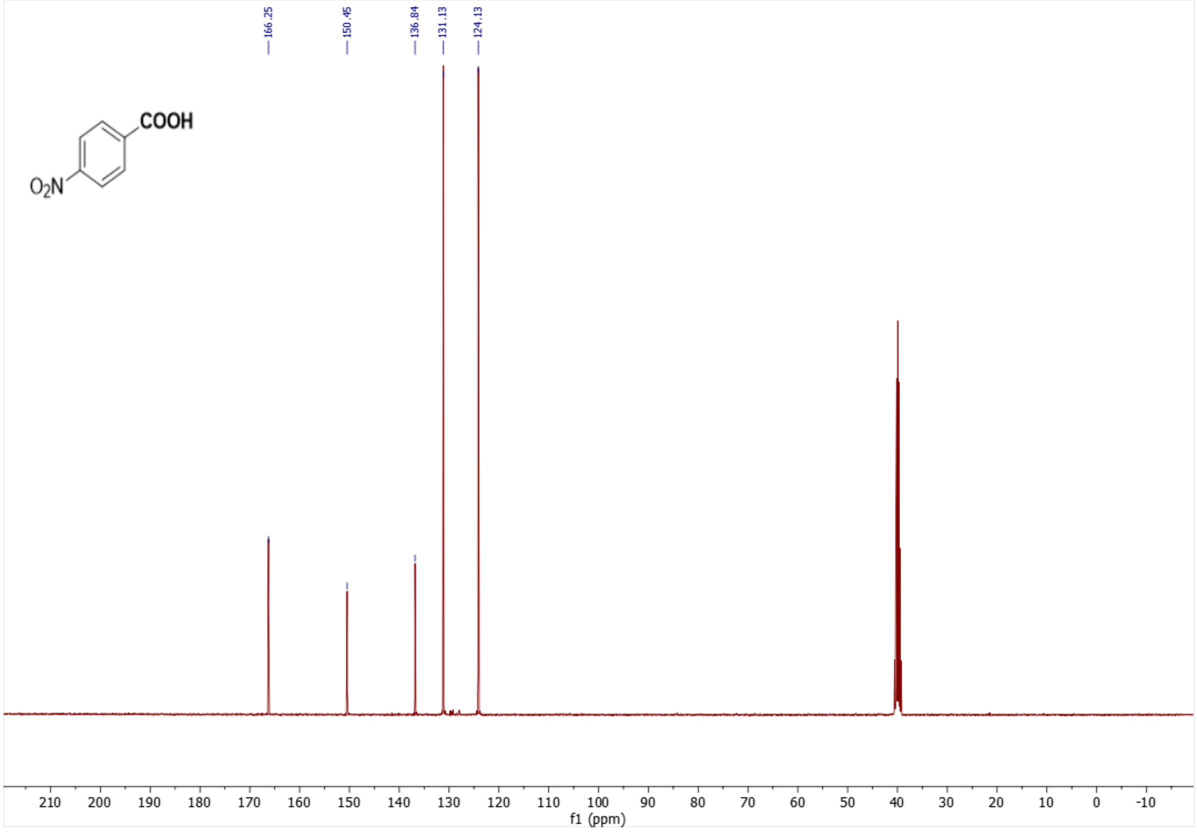

4-Cyanobenzoic acid (2k)

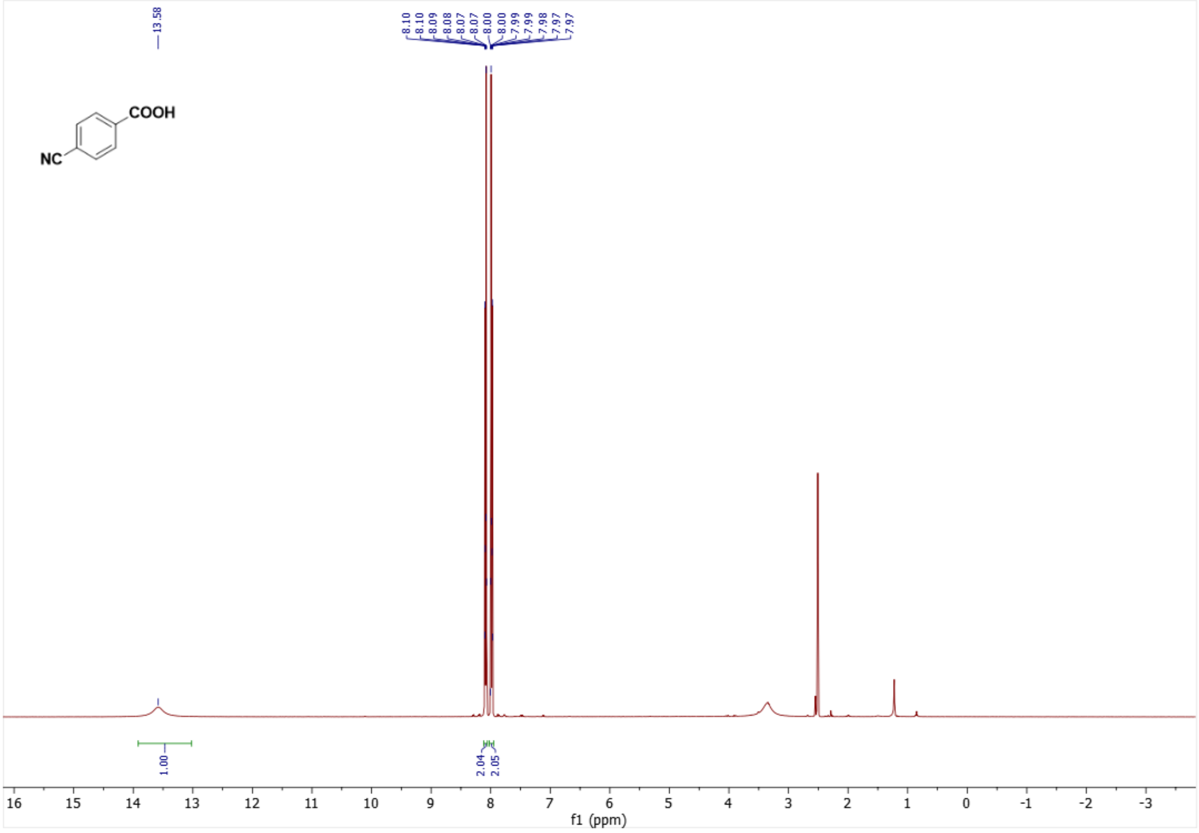

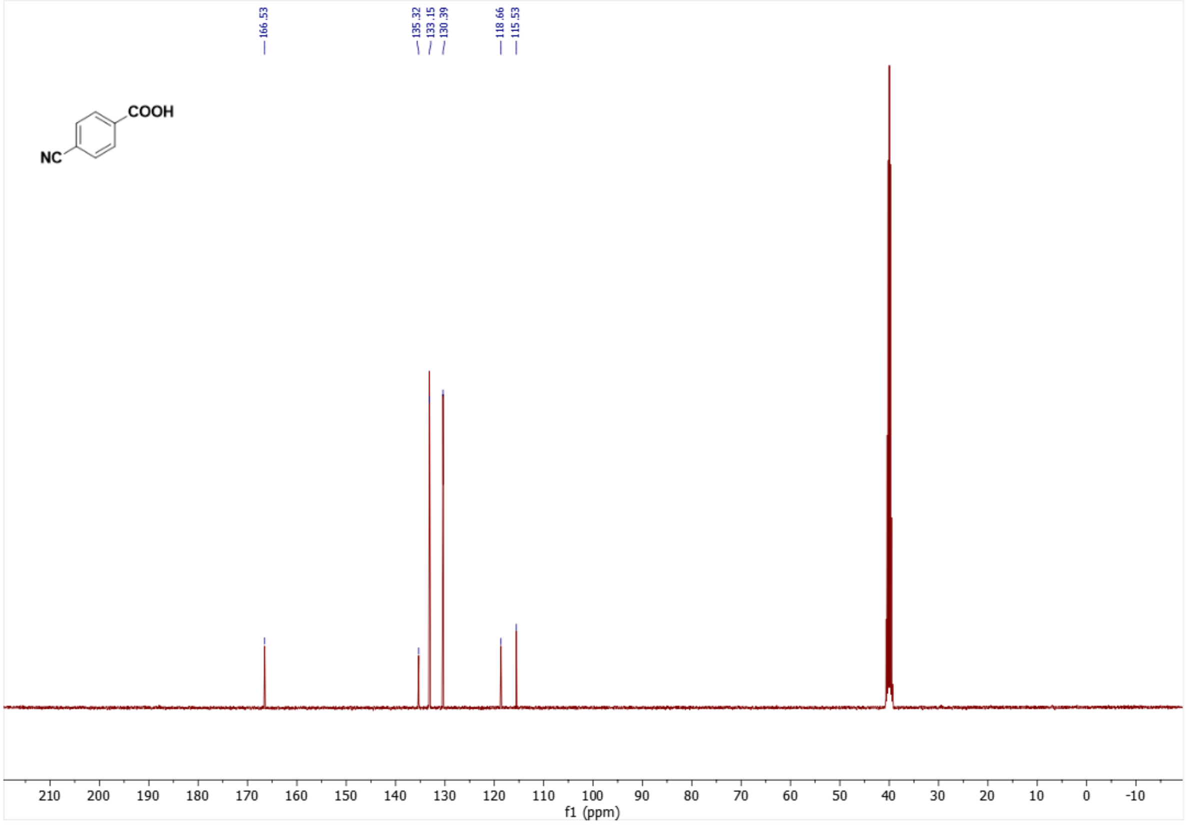

3-Nitrobenzoic acid (2l)

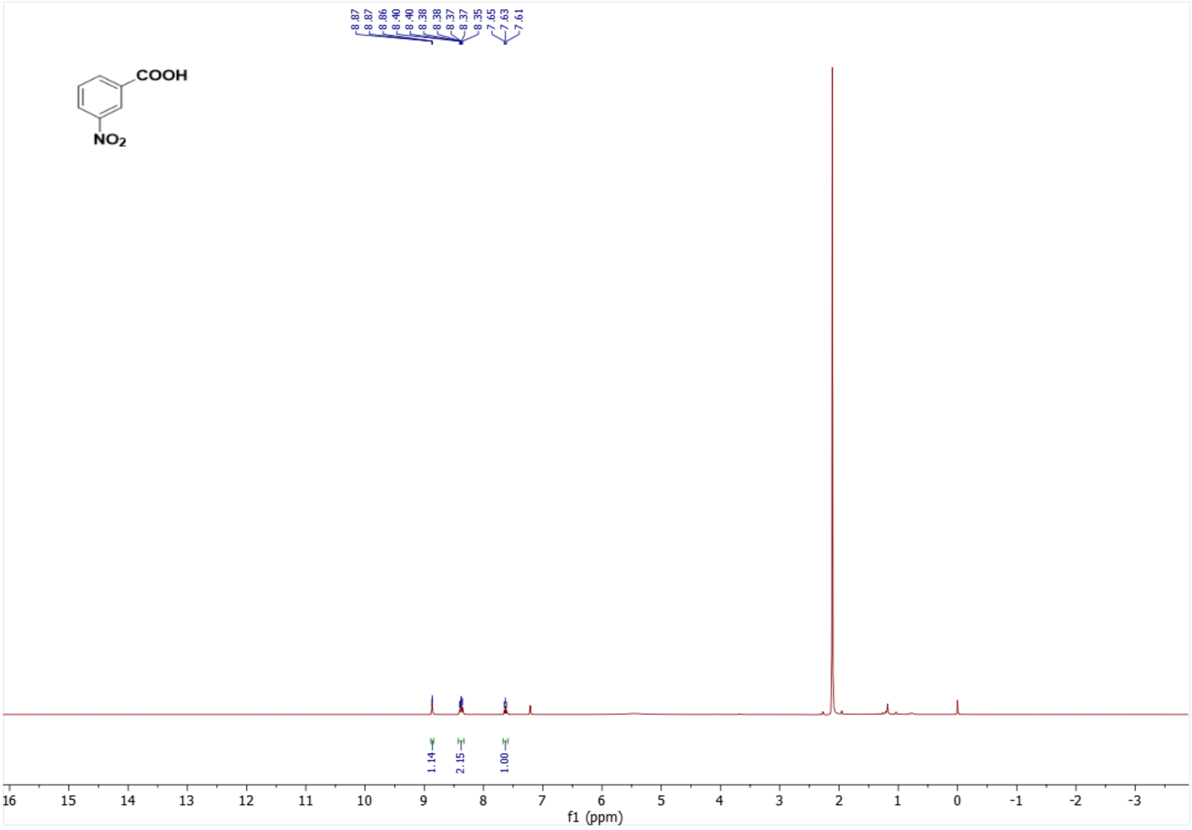

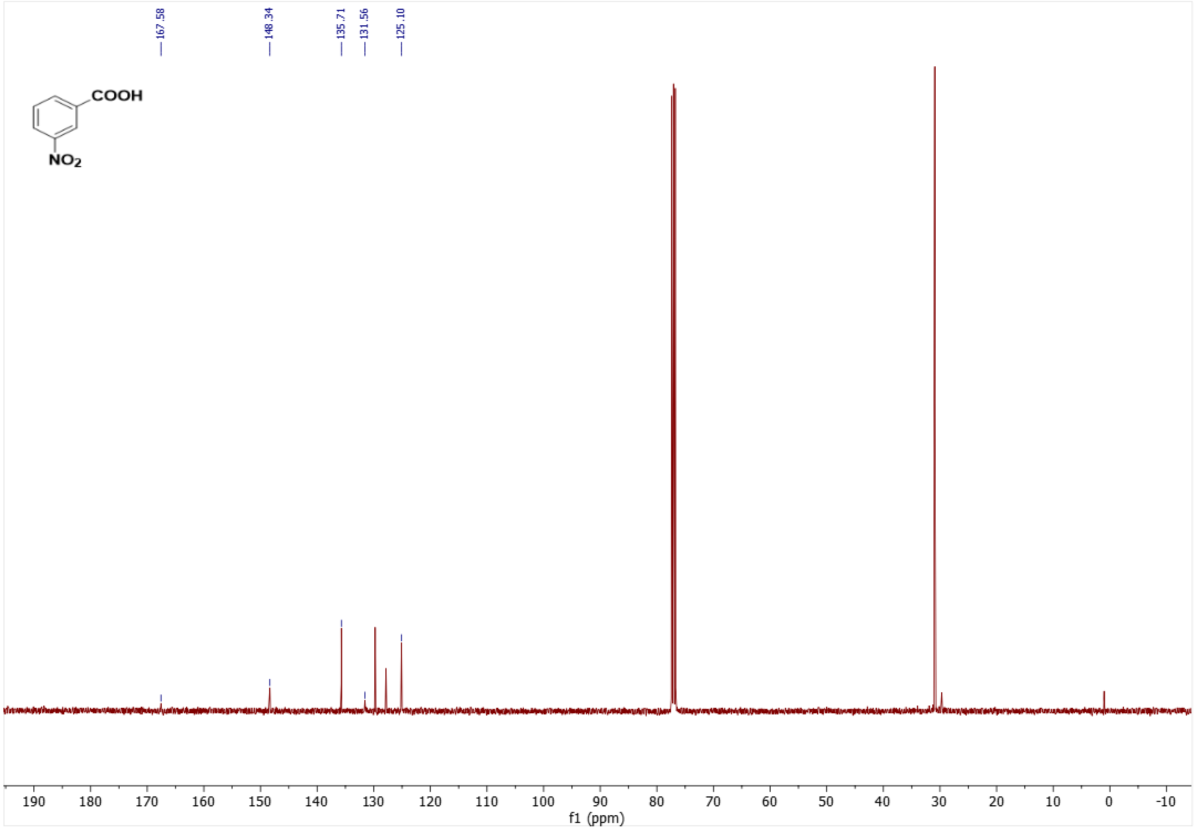

3-(Methoxycarbonyl)benzoic acid (2l’)

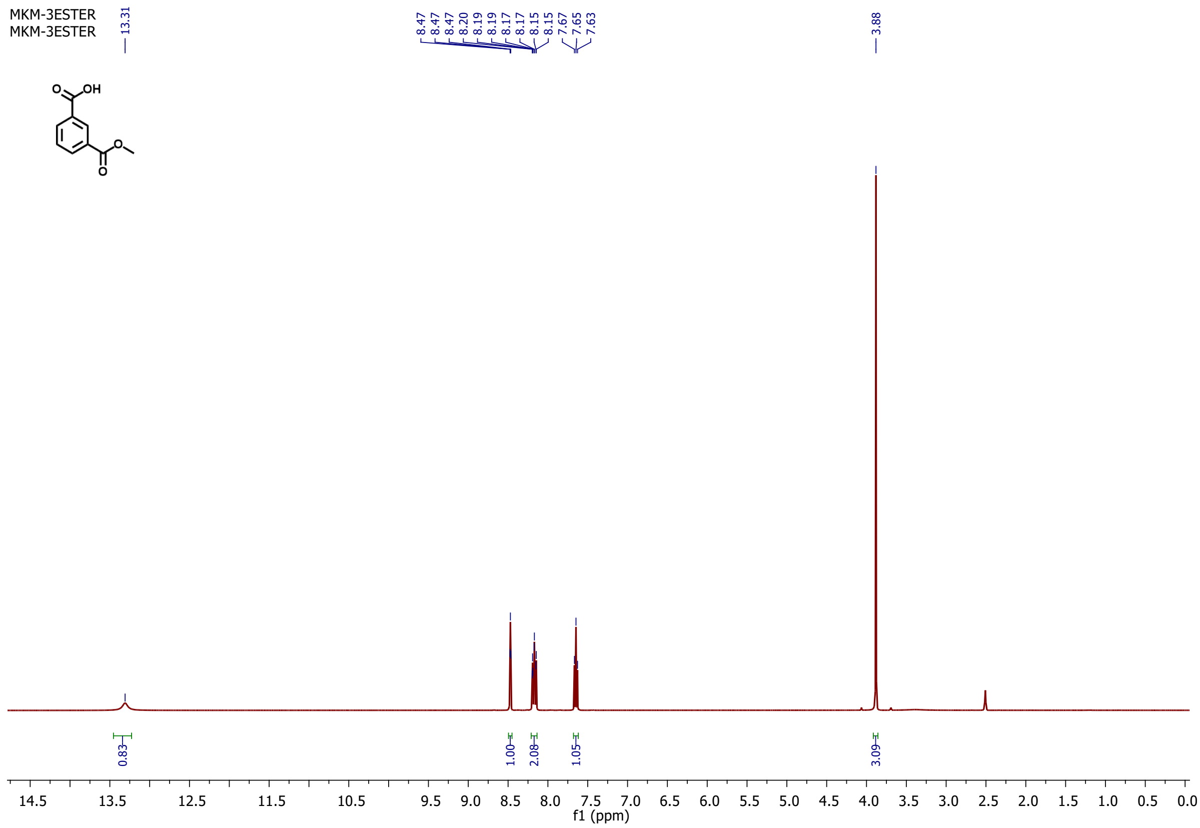

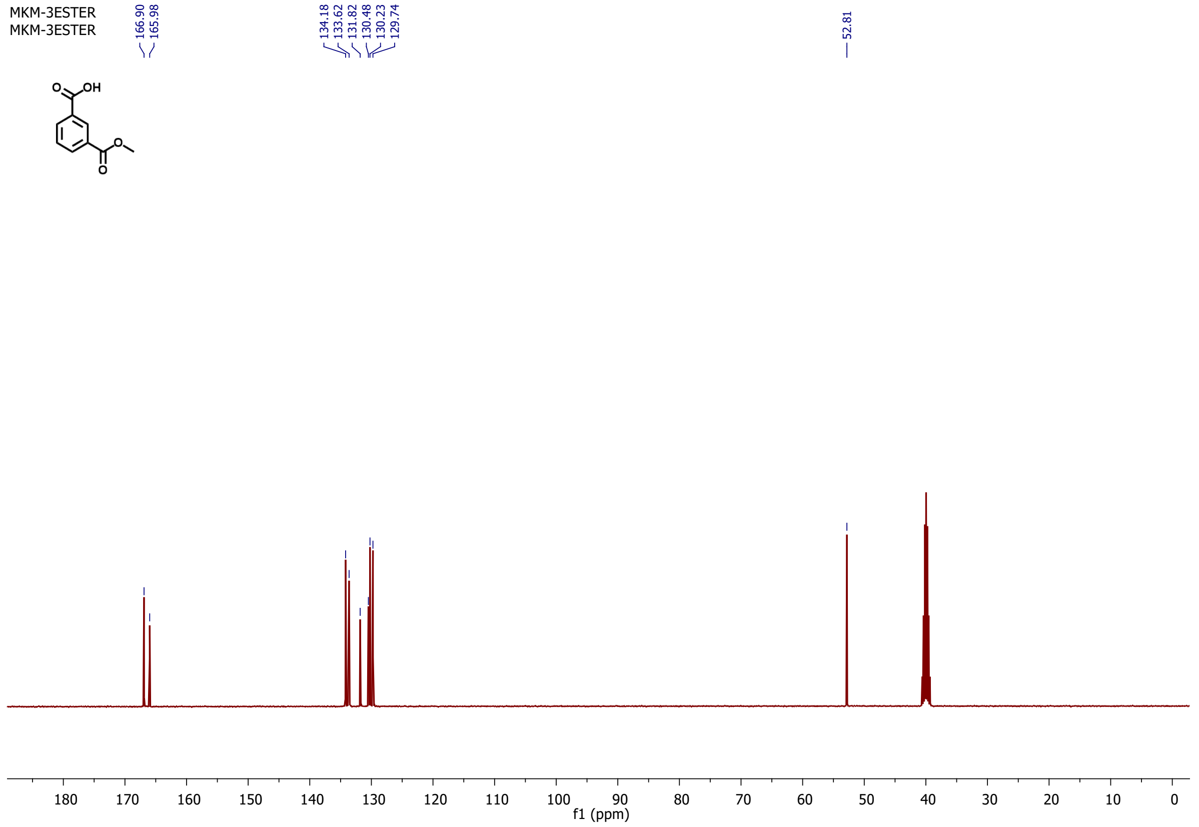

2-Hydroxybenzoic acid (2m)

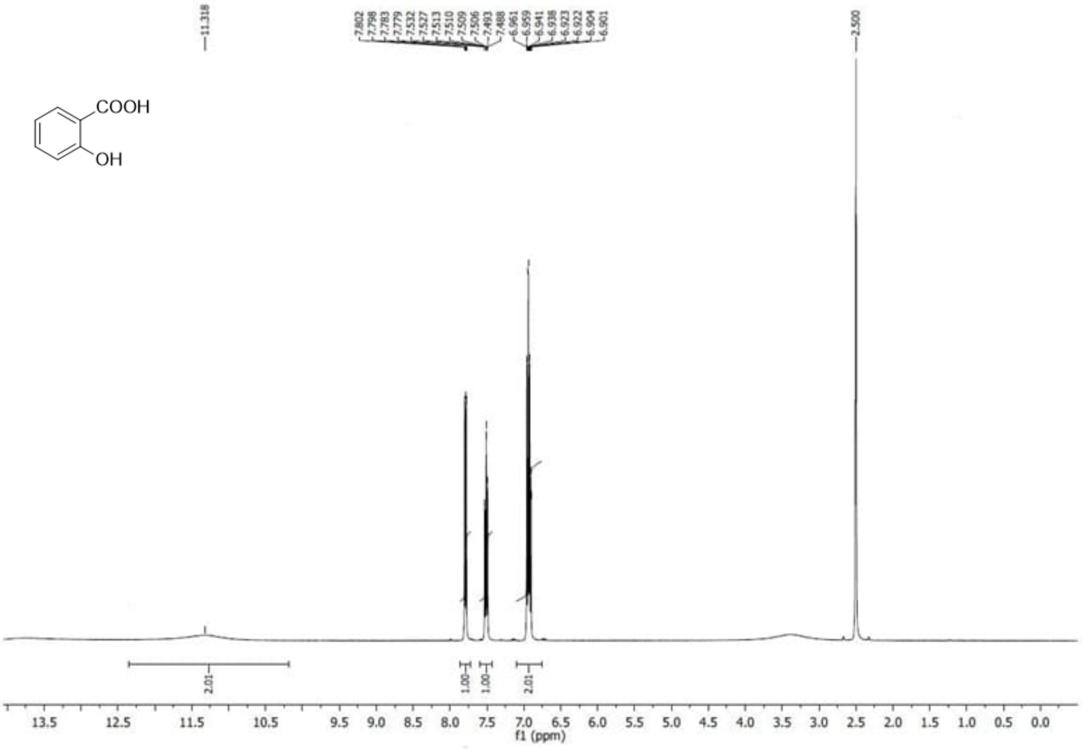

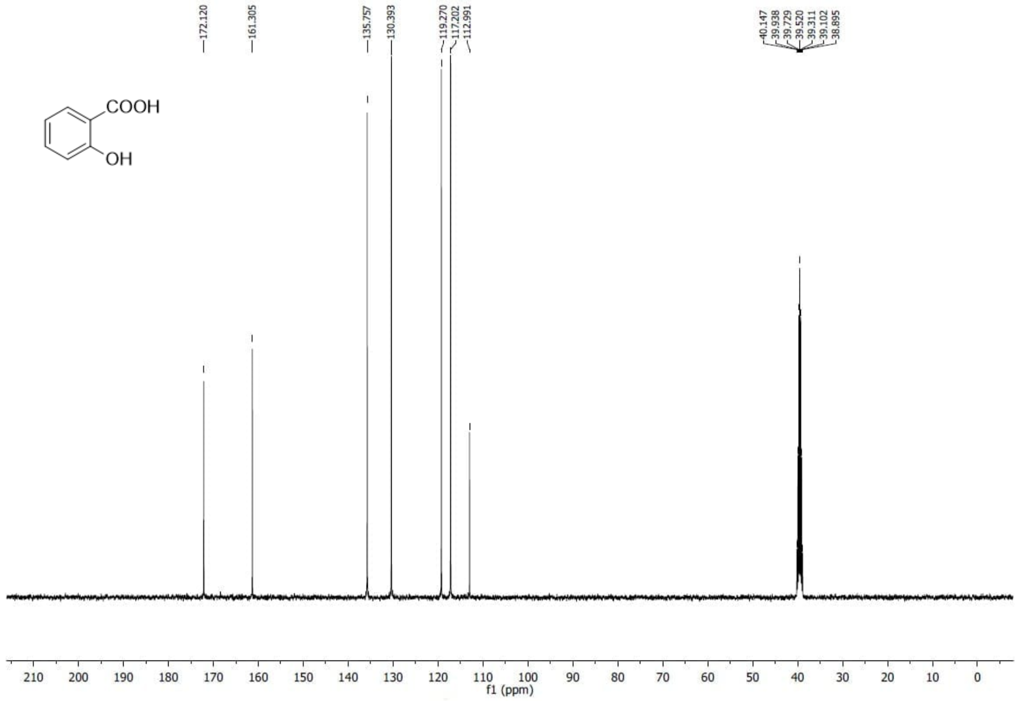

4-Hydroxybenzoic acid (2n)

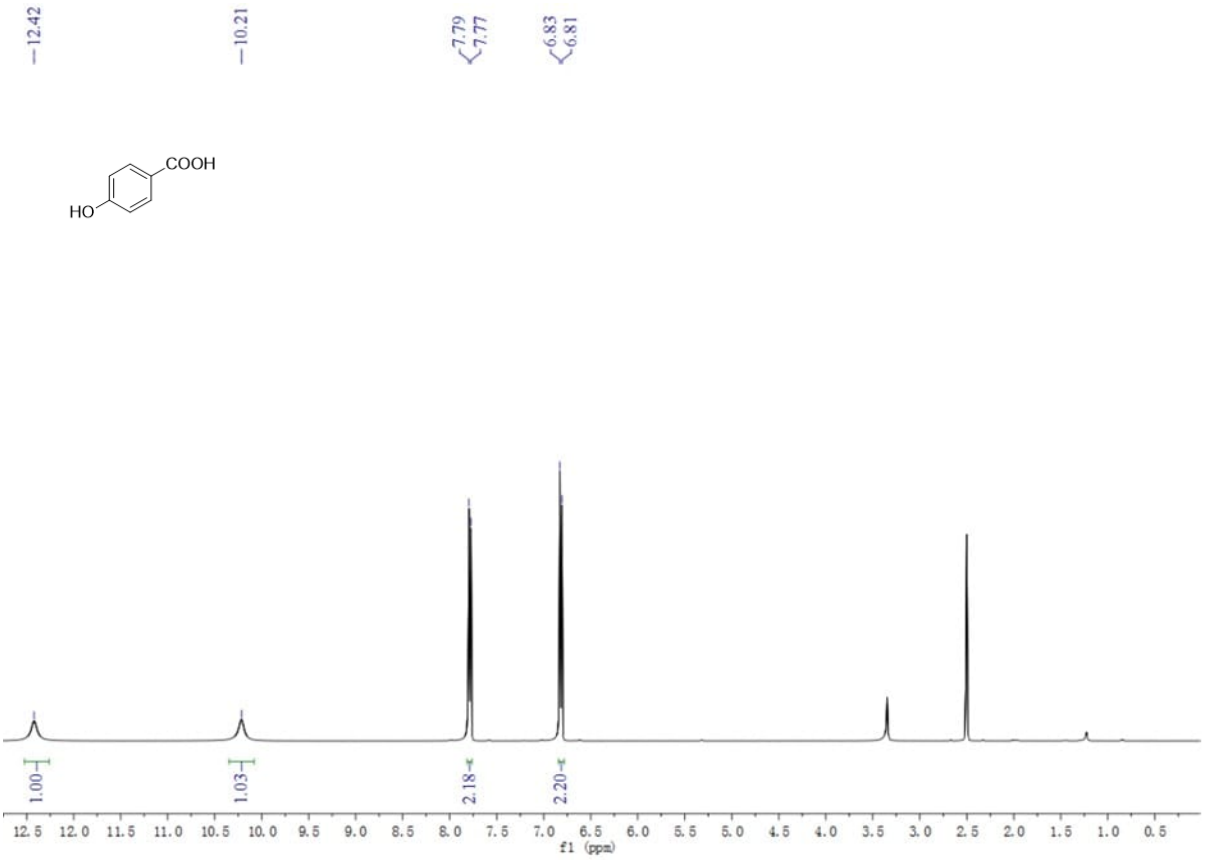

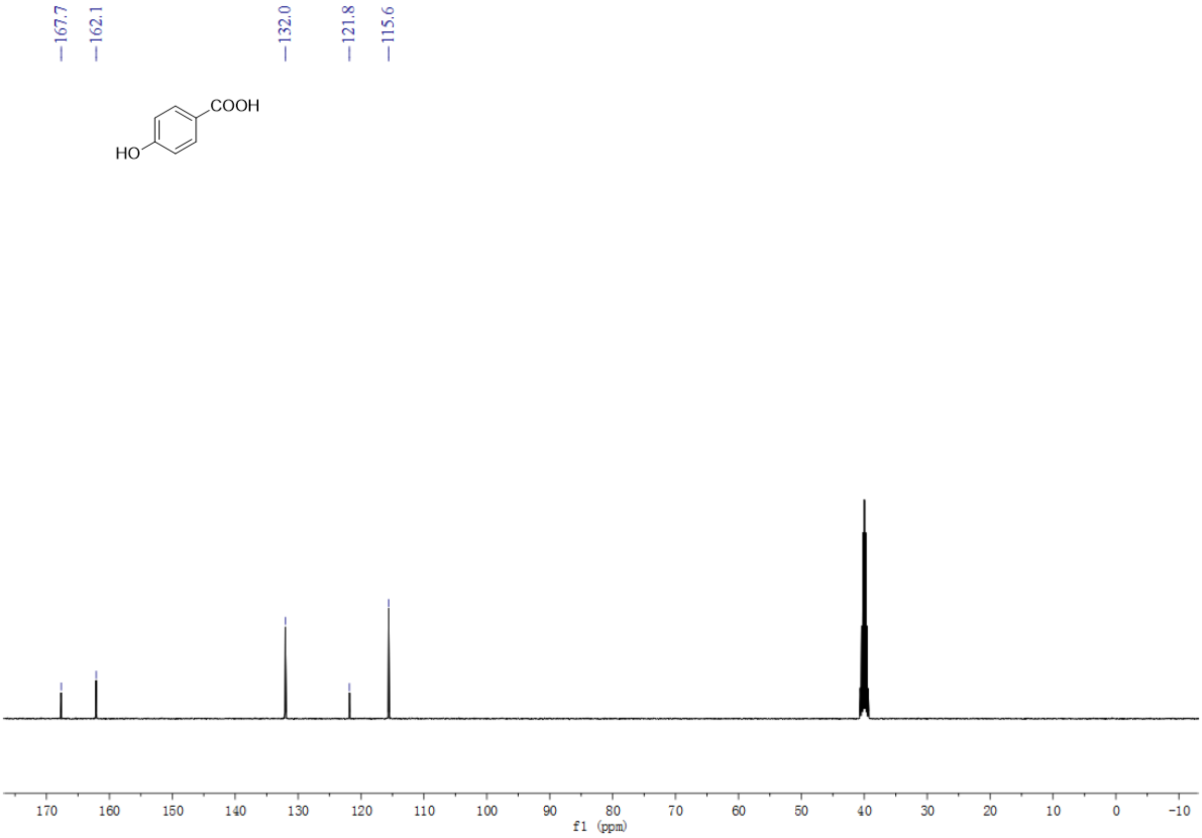

Thiophene-2-carboxylic acid (2o)

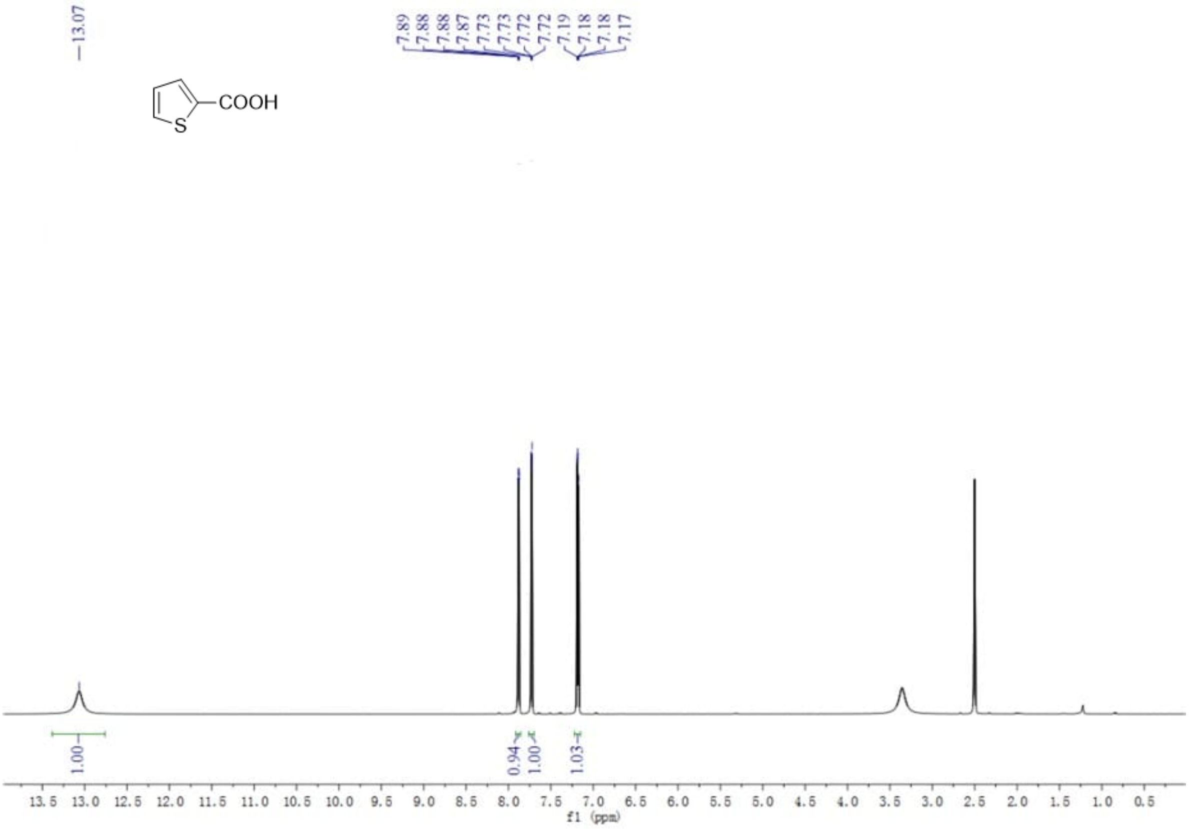

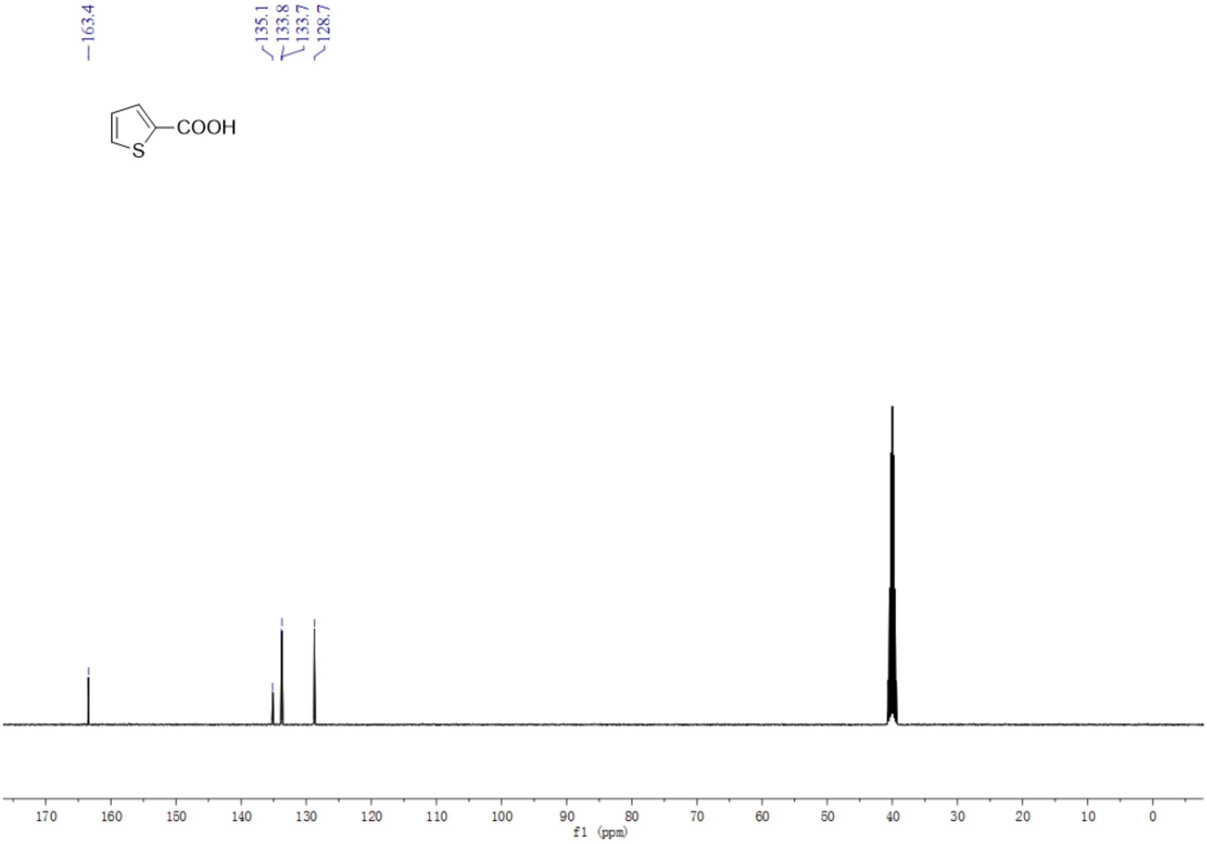

5. LCMS Spectra of crude reaction mixture of 1b

LCMS spectra for crude reaction mixture of reaction of **1b** under optimized conditions confirmed the release of *p*-toluenesulfinic acid confirmed by LCMS.

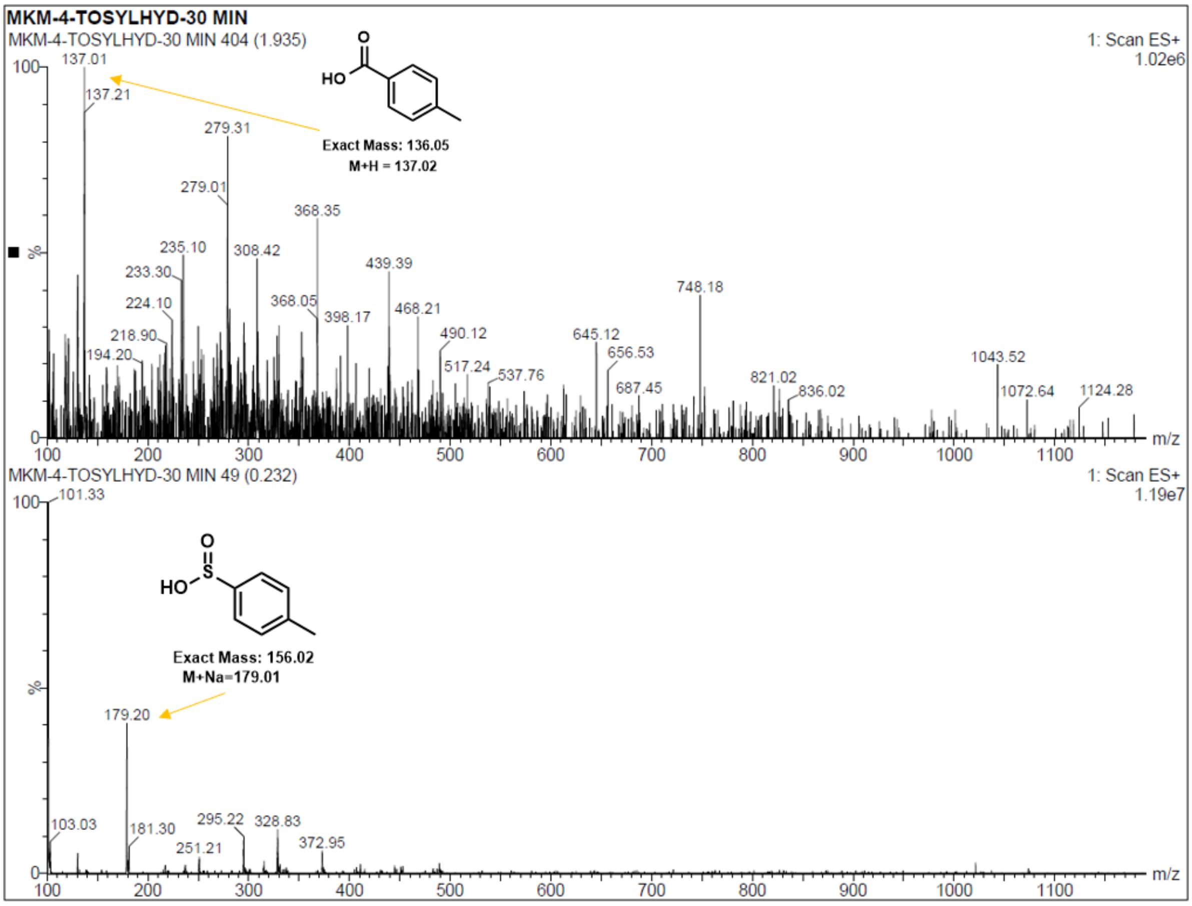

5. References

1. H. Yu, S. Ru, G. Dai, Y. Zhai, H. Lin, S. Han and Y. Wei, *Angew. Chem. Int. Ed.*, 2017, **56**, 3867-3871.
2. H. P. Kalmode, K. S. Vadagaonkar, S. L. Shinde and A. C. Chaskar, *J. Org. Chem.*, 2017, **82**, 3781-3786.
3. K.-J. Liu, Y.-L. Fu, L.-Y. Xie, C. Wu, W.-B. He, S. Peng, Z. Wang, W.-H. Bao, Z. Cao and X. Xu, *ACS Sustain. Chem. Eng.*, 2018, **6**, 4916-4921.
